# Restoring Compromised Cl^−^ in D2 Neurons of a Huntington’s Disease Mouse Model Rescues Motor Disability

**DOI:** 10.1523/JNEUROSCI.0215-24.2024

**Published:** 2024-11-05

**Authors:** Melissa Serranilla, Jessica C. Pressey, Melanie A. Woodin

**Affiliations:** Department of Cell and Systems Biology, University of Toronto, Toronto, Ontario M5S 3G5, Canada

**Keywords:** electrophysiology, GABA, Huntington’s disease, MSNs, synaptic inhibition

## Abstract

Huntington's disease (HD) is a progressive neurodegenerative disorder with no cure, characterized by significant neurodegeneration of striatal GABAergic medium spiny neurons (MSNs). Early stages of the disease are characterized by the loss of dopamine 2 receptor–expressing MSNs (D2 MSNs) followed by degeneration of dopamine 1 receptor–expressing MSNs (D1 MSNs), leading to aberrant basal ganglia signaling. While the early degeneration of D2 MSNs and impaired GABAergic transmission are well-documented, potassium chloride cotransporter 2 (KCC2), a key regulator of intracellular chloride (Cl^−^), and therefore GABAergic signaling, has not been characterized in D1 and D2 MSNs in HD. We aimed to investigate whether Cl^−^ regulation was differentially altered in D1 and D2 MSNs and may contribute to the early degeneration of D2 MSNs in male and female symptomatic R6/2 mice. We used electrophysiology to record the reversal potential for GABA_A_ receptors (*E*_GABA_), a read-out for the efficacy of Cl^−^ regulation, in striatal D1 and D2 MSNs and their corresponding output structures. During the early symptomatic phase (P55–P65), Cl^−^ impairments were observed in D2 MSNs in R6/2 mice, with no change in D1 MSNs. Cl^−^ regulation was also dysfunctional in the globus pallidus externa, resulting in GABA-mediated excitation. When we overexpressed KCC2 in D2 MSNs using AAV-mediated delivery, we delayed the onset of motor impairments in R6/2 mice. We demonstrate that Cl^−^ homeostasis is differentially altered in D1 and D2 MSNs and may contribute to the enhanced susceptibility of D2 MSNs during HD progression.

## Significance Statement

Huntington's disease is a neurodegenerative disease caused by a repeat expansion in the Huntingtin gene and characterized by the loss of dopamine 2 and dopamine 1 receptor–expressing medium spiny neurons [D2 and D1 medium spiny neurons (MSNs)] of the striatum. MSNs release GABA, which depends on proper Cl^−^ regulation for inhibition. We asked whether Cl^−^ homeostasis is differentially altered in D1 and D2 MSNs and their output structures and whether this altered expression contributes to the pattern of degeneration between these two cell types. Using electrophysiology, biochemistry, and fluorescence imaging, we determined that Cl^−^ regulation was impaired in D2 MSNs in R6/2 mice, with no change in D1 MSNs. Cl^−^ was also dysregulated in the globus pallidus externa resulting in excitatory GABA.

## Introduction

Huntington's disease (HD) is an inherited neurodegenerative disorder caused by a triple cytosine–adenine–guanine (CAG) repeat expansion in the gene encoding for the Huntingtin (Htt) protein (MacDonald et al., 1993). CAG repeat length determines the time of disease onset and severity (Snell et al., 1993; Lee et al., 2019). HD is characterized by progressive motor dysfunction, beginning with choreatic movements that later develop into hypokinesia and bradykinesia (Huntington, 1872). These biphasic changes in motor dysfunction are caused by differential susceptibility in the direct and indirect pathways of basal ganglia (BG) circuitry (Albin et al., 1992), which exert opposing effects on BG output. Activation of the direct pathway promotes movement while activation of the indirect pathway inhibits unwanted movements (Alexander and Crutcher, 1990). In HD, gamma-aminobutyric acid (GABA)-releasing medium spiny neurons (MSNs) of the indirect pathway, which primarily express the D2 dopamine receptor (D2 MSNs), demonstrate an enhanced susceptibility to neurodegeneration, leading to the development of chorea (Albin et al., 1992). At late stages of the disease, MSNs of the direct pathway which primarily express the D1 dopamine receptor (D1 MSNs) also degenerate, which ultimately leads to rigidity and a hypokinetic state (Thompson et al., 1988).

Impaired GABAergic inhibition underlying synaptic dysfunction is well-documented in HD (Cepeda et al., 2004, 2013; Holley et al., 2015; Indersmitten et al., 2015). In the mature central nervous system (CNS), synaptic inhibition is primarily mediated by GABA, which binds and opens GABA_A_ receptors (GABA_A_Rs) that are permeable to chloride ions (Cl^−^; Kaila et al., 1989). The strength and direction of Cl^−^ flow in neurons are determined by the intracellular Cl^−^ concentration ([Cl^−^]*_i_*), regulated primarily by the antagonistic actions of two cation–chloride cotransporters (CCCs): Cl^−^ -importing Na^+^–K^+^–Cl^−^ cotransporter 1 (NKCC1) and Cl^−^ -exporting K^+^–Cl^−^ cotransporter 2 (KCC2). In the immature brain, NKCC1 expression is higher than KCC2, leading to increased [Cl^−^]*_i_* and Cl^−^ efflux upon GABA_A_R activation. As the brain matures, KCC2 expression increases, resulting in lower levels of [Cl^−^]*_i_* and Cl^−^ influx upon GABA_A_R activation (Rivera et al., 1999). In certain neuropathological states, impaired KCC2 function and/or enhanced NKCC1 function can lead to intracellular Cl^−^ accumulation and GABA-mediated excitation (Ben-Ari et al., 2012); however, the potential role of impaired CCCs in the BG during early symptomatic HD is unknown.

KCC2 interacts with Htt (Shirasaki et al., 2012; Kalathur et al., 2015; Dargaei et al., 2018), and reports have linked altered CCC function to cognitive and behavioral impairments in HD, with inhibition of NKCC1 improving both cognitive (Dargaei et al., 2018) and motor (Hsu et al., 2019) deficits in various mouse models of HD. However, the role of CCCs has not been fully characterized in the direct and indirect pathways of the BG in the HD brain despite the well-documented susceptibility of D2 MSNs in HD. The selective loss of KCC2 in D2 MSNs has been previously linked to impaired locomotion (Barbour et al., 2021), lending support to the possibility of differential changes of KCC2 in the direct and indirect pathways of the BG underlying motor dysfunction.

Here, we used electrophysiology to assess CCC function in D1 and D2 MSNs in R6/2 mice during the early symptomatic phase. R6/2 is the first transgenic mouse model of HD; it contains exon 1 of the mutant HD gene and displays an aggressive phenotype with motor deficits at ∼P60 (Mangiarini et al., 1996). In early symptomatic HD mice (P55–P65), we demonstrate that Cl^−^ regulation is differentially impaired in the direct and indirect pathway of the BG and overexpression of KCC2 in D2 MSNs is sufficient to improve motor impairments, providing insight into enhanced vulnerability of D2 MSNs during HD progression.

## Materials and Methods

### Animals

All animal procedures were approved by the University of Toronto Animal Care Committee in accordance with the Canadian Council for Animal Care guidelines. All efforts were made to minimize animal suffering and to reduce the number of animals used. Mice were maintained in the Biological Sciences Facility in the Faculty of Arts and Science at the University of Toronto. Mice were group housed in a temperature-controlled room on a 12 h light/dark cycle with *ad libitum* access to food and water (3–4 mice per cage). Two HD mouse models were used in this study (obtained from The Jackson Laboratory): R6/2 mice containing the mutated Htt gene expressing exon 1 of the human Htt gene carrying ∼120 ± 5 CAG repeat expansions (Mangiarini et al., 1996) and YAC128 mice containing full-length human Htt with 128 CAG repeats (Slow et al., 2003). The R6/2 mice were maintained by crossing WT male C57BL/6×CBA mice with WT females that have transplanted R6/2 ovaries. Male and female YAC128 mice and their nontransgenic littermates were maintained on the FVB/N background. To differentiate D1 and D2 MSNs, DA D1-Cre and DA D2-Cre mice were used (also obtained from The Jackson Laboratory). D1 and D2 mice were crossed with WT female C57BL/6CBA mice transplanted with R6/2 ovaries to generate R6/2 and WT littermates expressing D1-Cre and D2-Cre. CAG repeat lengths for R6/2 averaged ∼120 CAG repeats. Genotype was determined twice: once at 2 weeks of age using PCR of tail snips and again after being killed; PCR was performed by the Centre for Applied Genomics at the Research Institute of the Hospital for Sick Children (Toronto, Canada). Both males and females were used for all experiments.

To express mCherry and KCC2 in D1 and D2 MSNs in WT and R6/2 mice, D1-Cre and D2-Cre mice were crossed to R6/2 females. AAV5-hSyn -DIO-mCherry (Addgene plasmid #50459; 250 nl/site, 7 × 10^12^ vg/ml, 0.1 µl/min) or AAV5-hSyn-FLEX-KCC2(HA) (250 nl/site, 6 × 10^12^ vg/ml, 0.1 µl/min; Canadian Neurophotonics Platform) was stereotaxically injected into the striatum of P18–P20 WT and R6/2 mice crossed with D1-Cre and D2-Cre mice (C57BL/6J background) with the following coordinates: 0.75 mm anterior and 1.75 mm lateral to bregma at a depth of 2.70 mm from the dura. For P35–P40 mice, the following coordinates were used: 1.0 mm anterior and 2.0 mm lateral to bregma, at a depth of 3.1 mm from the dura. The expression of these proteins was visualized by mCherry fluorescence or HA expression after behavioral assays were performed.

### Mouse brain slice preparation

Mice were deeply anesthetized with a mix of ketamine (150 mg/kg) and xylazine (15 mg/kg) and perfused with ice-cold NMDG–HEPES–aCSF solution containing the following (in mM): 92 NMDG, 2.5 KCl, 1.25 NaH_2_PO_4_, 30 NaHCO_3_, 20 HEPES, 25 glucose, 2 thiourea, 5 Na-ascorbate, 3 Na-pyruvate, 0.5 CaCl_2_, and 10 MgCl_2_ in double-distilled water (ddH_2_O) saturated with 95% O_2_/5% CO_2_, adjusted to pH 7.4 with HCl, and an osmolarity of 300–305 mOsm. Brains were rapidly removed after decapitation and placed into prechilled NMDG–HEPES– aCSF. Coronal striatal slices (300 µm) were prepared from P55–P65 R6/2 mice or 1-year-old YAC128 mice and their WT littermates using a Compresstome VF-300-0Z (Precisionary Instruments). Slices were transferred into a prewarmed (33–34°C) chamber filled with NMDG–HEPES–aCSF for initial recovery for ∼12–13 min and then transferred to a recovery solution at room temperature (RT) containing HEPES holding aCSF (in mM): 92 NaCl, 2.5 KCl, 1.25 NaH_2_PO_4_, 30 NaHCO_3_, 20 HEPES, 25 glucose, 2 thiourea, 5 Na-ascorbate, 3 Na-pyruvate, 2 CaCl_2_·2H_2_O, and 2 MgSO_4_·7H_2_O and adjusted pH to 7.3–7.4 with NaOH in ddH_2_O saturated with 95% O_2_/5% CO_2_, adjusted to pH 7.4 with NaOH, and an osmolarity of 300–305 mOsm for an hour. All chemicals were purchased from Sigma-Aldrich unless otherwise specified.

### Electrophysiology

Recordings were obtained from MSNs in the dorsolateral striatum due to their involvement in regulating motor behavior (Robbins and Everitt, 1996; Groenewegen, 2003; Haber et al., 2012). MSNs were identified by their medium-sized somas, a hyperpolarized resting membrane potential (RMP) more negative than −70 mV, the presence of a small depolarizing sag, and prominent inward rectification in response to hyperpolarizing current pulses (Belleau and Warren 2000). Recording pipettes were pulled from thin-walled borosilicate glass pipettes (TW-150 F, World Precision Instruments) to resistances of 5−7 MΩ with a Sutter Instrument P-87. Micropipettes were filled with an intracellular solution (ICS) containing the following (in mM): 130 K^+^-gluconate, 10 KCl, 10 HEPES, 0.2 EGTA, 4 ATP, 0.3 GTP, and 10 phosphocreatine, pH 7.4 with KOH and osmolarity 300 mOsm. For Cl^−^ loading experiments in whole-cell configuration, pipettes were filled with ICS the following (in mM): 110 K^+^-gluconate, 30 KCl, 10 HEPES, 0.2 EGTA, 4 ATP, 0.3 GTP, and 13 phosphocreatine, pH 7.4 with KOH and osmolarity 300 mOsm. For perforated patch recording, we used a pipette solution containing the following (in mM): 150 KCl and 10 HEPES. Gramicidin was dissolved in DMSO at 60 mg/ml and added to the pipette solution to make a final concentration of 30–60 µg/ml. The electrode tip was filled with gramicidin-free solution and the barrel with gramicidin-containing solution. Recordings were made in standard aCSF containing the following (in mM): 123 NaCl, 2.5 KCl, 1.25 NaH_2_PO_4_, 25 NaHCO_3_, 25 glucose, 2 CaCl_2_, and 1 MgCl_2_ in ddH_2_O, saturated with 95% O_2_/5% CO_2_, pH 7.4 with NaOH and an osmolarity of 300–305 Osm. Whole-cell recordings were initiated 10 min after membrane rupture. Signals were amplified using an Axon Instruments MultiClamp 700B and digitized using an Axon Instruments Digidata 1550b (Molecular Devices). To determine *E*_GABA_, neurons were held at −70 mV under a whole-cell voltage clamp, and the membrane potential was stepped in +10 mV increments from −100 to 0 mV (a smaller subset of these step potentials are presented in representative traces in figures). During each membrane potential step, local electrical stimulation was used to activate inhibitory postsynaptic currents (IPSCs) in the presence of 6-cyano-7-nitroquinoxaline-2,3-dione (CNQX; 10 µM) and 2-amino-5-phosphonopentanoic acid (AP-V; 50 µM) to block glutamatergic transmission. To assess *E*_GABA_ after NKCC1 inhibition, bumetanide (10 µM) was perfused into the recording chamber. *E*_GABA_ was recorded before and 20 min after bumetanide bath application. The maximum current amplitude was taken as the largest absolute current recorded during the recordings performed for the *E*_GABA_ measurement. Using Clampfit 10.7 (Molecular Devices), two cursors were placed on the resulting recording trace (one just before the current and the other at the peak of the current), and the peak amplitude was exported to Prism (version 9) and graphed against the holding potential. A linear regression of the IPSC amplitude versus membrane potential was achieved using Prism, the intercept of this line with the abscissa was taken as *E*_GABA_, and the slope of this line divided by the capacitance was taken as the synaptic conductance density. The theoretical value of *E*_GABA_ was calculated ([Cl^−^]*_i _*= 10 mM, *E*_GABA _= −65 mV; [Cl^−^]*_i_* = 30 mM, *E*_GABA _= −37 mV) using the Nernst equation and compared with experimental values of *E*_GABA_ to determine the efficacy of Cl^−^ extrusion. For RMP, a whole-cell patch clamp was achieved, the amplifier was set to *I* = 0, and the corresponding potential was measured under current-clamp mode. Values have not been corrected for whole cell for the liquid junction potential of 3.3 mV for 10 mM Cl^−^ and 3.9 mV for 30 mM Cl^−^. To record spiking activity induced by bath application of GABA, a cell-attached voltage-clamp configuration was used. Micropipettes were filled with aCSF, and slices were bath perfused with aCSF. After a giga-ohm seal had been obtained, the following recordings were initiated for 5 min: baseline in aCSF, GABA (100 µM), GABA 100 µM + gabazine (10 µM). The recording pipette was kept at 0 mV under a voltage clamp for all recordings. Data from the whole 5 min of recording were analyzed to quantify spiking activity. Data were extracted using threshold-based detection using Clampfit 10.7 (Molecular Devices). The dependence of the spiking activity on voltage-gated Na^+^ channels was confirmed by TTX application at the end of a subset of the recordings.

### Biochemistry

For protein extraction, the dorsolateral striatum was microdissected from R6/2 and WT samples on ice in 1× phosphate-buffered saline (PBS) and was homogenized using a glass Teflon homogenizer followed by a brief low-speed centrifugation. Soft pellets were resuspended in ice-cold lysis buffer (4× weight/volume) containing the following (in mM): 50 Tris-HCl pH 7.4, 150 NaCl, 0.05 EDTA, and protease and phosphatase inhibitor mixture (Roche), homogenized and centrifuged for 30 min at 25,000 × *g*. The membrane pellets were resuspended in solubilization buffer (4× weight/volume) containing the following (in mM): 50 Tris·HCl pH 7.4, 150 NaCl, 0.05 EDTA, 1.5% C_12_E_9_, and protease and phosphatase inhibitor mixture, solubilized for 4 h on a rotating platform and centrifuged for 1 h at 25,000 × *g*. Protein concentration was determined using a protein assay kit (Bio-Rad). All biochemical preparations and centrifugations were performed at 4°C. For immunoblot analysis, equal amounts of protein (15 µg) were run on 7% Tris-HCl SDS–PAGE gels and transferred onto nitrocellulose membranes (GE HealthCare). Membranes were probed with anti-NKCC1 (1:1,000, clone T4, Developmental Studies Hybridoma Bank), anti-KCC2 (1:1,000, 07-432, EMD Millipore), and anti β-actin (1:2,000, 4967S, Cell Signaling Technology), followed by HRP-conjugated secondary antibodies (7074, 7076, Cell Signaling Technology). Chemiluminescence signals were digitally acquired using ChemiDoc XRS+ (Bio-Rad), and the band intensities were quantified using Image Lab (Version 5.2.1, Bio-Rad).

### Immunostaining and confocal imaging

Mice were transcardially perfused with ice-cold 1× PBS, followed by 4% PFA. Brains were extracted and postfixed in 4% PFA overnight at 4°C and cryoprotected in 30% sucrose and stored at −80°C. Striatal slices were cut coronally at 30 µm thickness on a Leica cryostat (CM 1520). For KCC2 or HA staining, free-floating striatal sections were rinsed once in 1× PB for 10 min three times. Slices were then blocked in 1× PB containing 5% BSA and 0.5% Triton X-100 for 3 h at RT followed by overnight incubation at 4°C in 1× PB with 0.1% Triton X-100 with rabbit polyclonal anti-KCC2 (1:1,000, ab97502, Abcam) and mouse monoclonal anti-HA (1:1,000, ROAHA, Roche) at 4°C. Finally, slices were incubated in Alexa Fluor 555–conjugated goat anti-rabbit antibody (A-21428, Thermo Fisher Scientific) for KCC2 and Alexa Fluor 488–conjugated goat anti-mouse (A-11029, Thermo Fisher Scientific) for HA for 2 h at RT. For A2AR and substance P staining, free-floating striatal sections were rinsed once in 1× PB for 15 min three times. After antigen retrieval in a solution of sodium citrate (50 mM, for 30 min at 80°C), the slices were incubated in primary antibodies for 24 h at RT, followed by 48 h at 4°C. Rabbit polyclonal antibody for A2AR (1:250, BML-SA654-0200, Enzo Life Sciences) and rabbit polyclonal antibody for substance P (1:200, AB1566, Millipore) were diluted in 0.1 M PB containing 0.3% (v/v) Triton X-100 and 0.02% NaN3. Sections were rinsed in 1× PB for 15 min three times and incubated at 4°C for 48 h with Alexa Fluor 555–conjugated goat anti-rabbit antibody (A-21428, Thermo Fisher Scientific) for substance P and Alexa Fluor 488–conjugated goat anti-rabbit antibody (A-11008, Thermo Fisher Scientific) for A2AR for 2 h at RT. Mounted slices were imaged on a Leica TCS SP8 confocal system with a Leica DMI 6000 inverted microscope (Quorum Technologies). Images were acquired using 3D image analysis software (Perkin Elmer). Images were obtained using a 20×, 40×, and 60× oil immersion objective. Imaging experiments were performed and analyzed in a blinded manner. Using ImageJ, four bisecting lines were drawn across the center of the cell. The peak values of each line (two values/line) were used to calculate the peak fluorescent intensity of KCC2 at the membrane.

### Bumetanide treatment

R6/2 and WT littermates were randomly assigned to bumetanide (Sigma-Aldrich; 0.2 mg/kg body weight) or vehicle groups (2% DMSO in saline) and treated daily by intraperitoneal injection from 7 weeks of age until 12 weeks of age. On the day of behavioral testing, injections were given at least 1 h before the beginning of the task.

### Rotarod

Rotarod performance was used to assess sensorimotor coordination and motor learning. For bumetanide therapy: Animals were trained for 1 week prior and tested at a constant speed of 10 rpm for 120 s. The latency to fall was recorded across three sessions per week and three trials per session; the average of the three trials was presented daily. Mice that could not perform greater than 60 s (50% of the maximal time) on the rotarod during Week 2 were omitted.

For overexpression of KCC2 in D1-Cre x R6/2 and D2-Cre x R6/2 mice: For the first week of testing, animals were trained using an accelerated rotarod protocol. Mice were placed on a rotating rod that accelerated quickly from 0–5 rpm and then gradually from 5–20 rpm. The following week, mice were tested with a constant speed of 10 rpm for 300 s. The latency to fall was recorded across two sessions per week and three trials per session; the average of the three trials was presented daily. Mice that could not perform greater than 150 s (50% of the maximal time) on the rotarod during Week 2 were omitted.

### Statistical analyses

Values shown are mean ± SD. Statistical significance was determined using statistical tests detailed in both the figure legends and the Results section. These tests included the following: Student's paired and unpaired two-tailed *t* tests (when normally distributed as assessed by the D'Agostino–Pearson; if *n* was too small, then Shapiro–Wilk test was used and when there were no differences in SD), unpaired two-tailed *t* tests with Welch's correction (when normally distributed but SDs were unequal), and two-way ANOVA followed by Tukey's multiple-comparisons tests. All *n* values are reported in the figure legends. Statistical significance was determined using Prism GraphPad software (Version 9.3.0). Statistical significance was determined as follows: *p < 0.05, **p < 0.01, ***p < 0.001.

## Results

### CCC protein expression is altered in the striatum of symptomatic R6/2 mice

**Figure 1.**
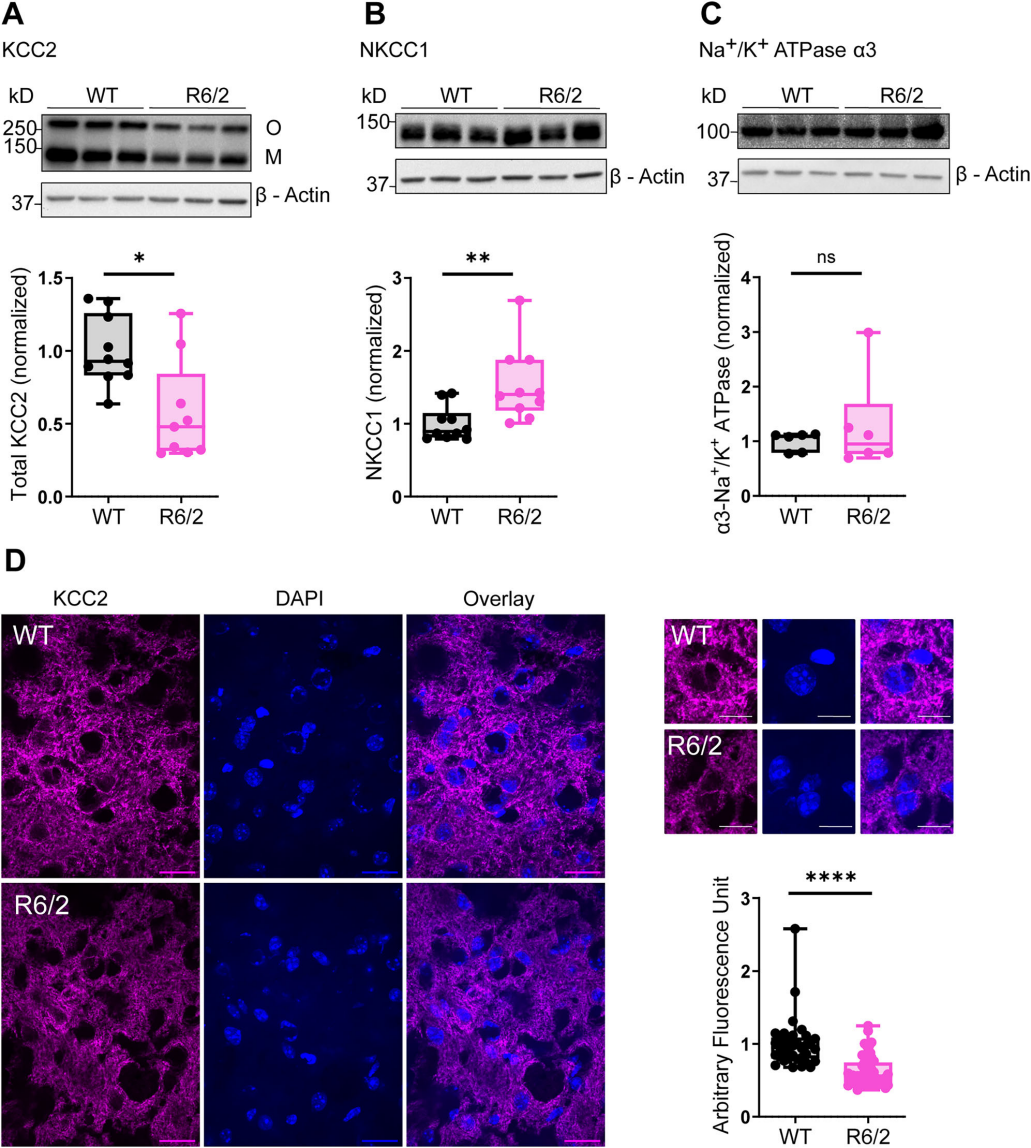
CCC protein expression is altered in the striatum of symptomatic R6/2 mice. ***A***, Top, Representative immunoblot images for KCC2 in protein extracts from samples of striatal lysates from WT and R6/2 mice. For full blots, see Extended Data Figure 1-1*A*. ***A***, Bottom, Quantification of total [oligomer (O) + monomer (M)] in WT (*N* = 10) and R6/2 (*N* = 9) normalized to β-actin (*p* = 0.0172; Mann–Whitney test). ***B***, Top, Representative immunoblot images for NKCC1 in protein extracts from samples of striatal lysates from WT and R6/2 mice. For full blots, see Extended Data Figure 1-1*B*. ***B***, Bottom, Quantification of NKCC1 in WT (*N* = 10) and R6/2 (*N* = 10) normalized to β-actin (*p* = 0.0029; Mann–Whitney test). ***C***, Top, Representative immunoblot images for Na^+^/K^+^ ATPase in protein extracts from samples of striatal lysates from WT and R6/2. For full blots, see Extended Data Figure 1-1*C*. ***C***, Bottom, Quantification of Na^+^/K^+^ ATPase in WT (*N* = 6) and R6/2 mice (*N* = 6) normalized to β-actin (*p* > 0.9999; Mann–Whitney test). For panels ***A–C***, the circles indicate the values from individual animals. The whiskers indicate min to max, and the line indicates the median. ***D***, Left, Representative confocal images of striatal sections from WT (top) and symptomatic R6/2 (bottom) mice labeled for KCC2 (magenta; scale bar, 20 µm). ***D***, Top right, Representative pictographs showing KCC2 expression (magenta; scale bar, 10 µm). ***D***, Bottom right, Summary quantification of KCC2 labeling in WT (*N* = 5, *n* = 36) and R6/2 (*N* = 5, *n* = 57; *p* < 0.0001; Mann–Whitney test). The circles indicate the values from single samples. The Whiskers indicate min to max, and the line indicates the median. For CCC protein expression in presymptomatic R6/2 and WT, see Extended Data Figure 1-2*A*–*E*.

During advanced stages of the disease, HD mice have decreased KCC2 mRNA and protein expression (Hsu et al., 2017). However, it is unknown whether KCC2 downregulation occurs at the early stages of the symptomatic phase and contributes to altered Cl^−^ homeostasis and the enhanced susceptibility of D2 MSNs and their synaptic dysfunction. To test this hypothesis, we performed Western blots using striatal lysates of R6/2 mice and their WT littermates at P60, the age at which motor dysfunction is first observed in this mouse model (Li et al., 2005). This analysis revealed a significant reduction in KCC2 protein expression (Extended Data Fig. 1-1*A*, Fig. 1*A*; *p* = 0.0172, Mann–Whitney test) accompanied by a significant increase in NKCC1 expression (Extended Data Fig. 1-1*B*, Fig. 1*B*; *p* = 0.0029, Mann–Whitney test) in the striatum of symptomatic R6/2 mice compared with their WT littermates, with no change in the control, Na^+^/K^+^ ATPase (Extended Data Fig. 1-1*C*, Fig. 1*C*; *p* > 0.9999, Mann–Whitney test). This decrease in KCC2 expression was also observed in striatal neurons using immunofluorescence (Fig. 1*D*; *p* < 0.0001, Mann–Whitney test). In addition, presymptomatic mice (P35–P38) exhibited a decreased ratio of KCC2 oligomers to monomers (Extended Data Fig. 1-2*E*; *p* = 0.0281, unpaired *t* test with Welch's correction), indicating altered oligomer/monomer ratio compared with WT littermates. Thus, decreased KCC2 expression in the striatum occurs early during disease progression in R6/2 mice.

10.1523/JNEUROSCI.0215-24.2024.f1-1 Figure 1-1 Full-size immunoblots related to Fig.1A-C used for quantification. O: Oligomer, M: Monomer. Yellow boxes indicate regions shown in the corresponding Figure. Download Figure 1-1, TIF file.

**Figure 2.**
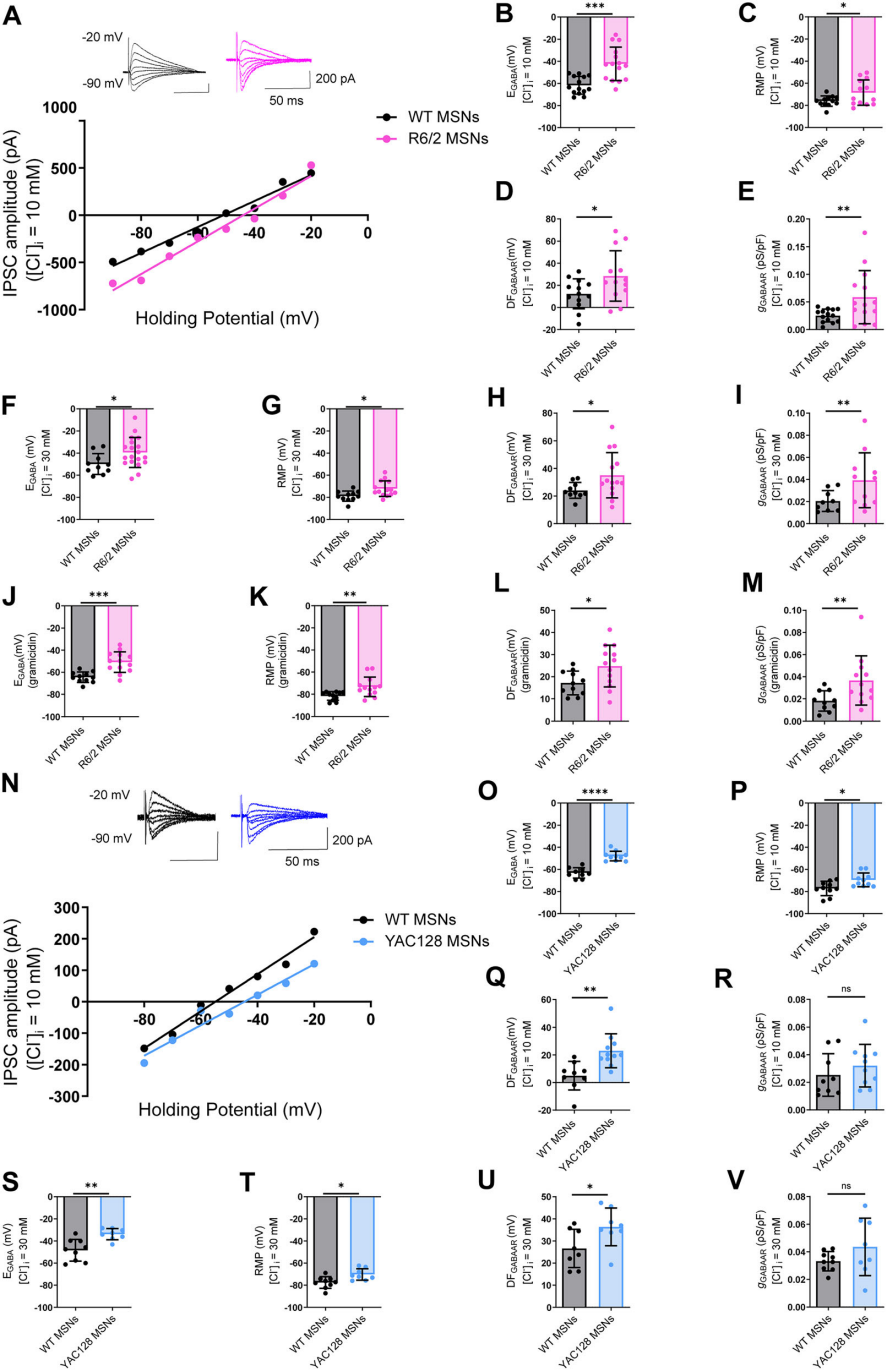
*E*_GABA_ is altered in striatal MSNs of symptomatic HD mice. ***A***, Example IV curves of representative IPSCs (inset) induced by electrical stimulation in the presence of glutamate blockers CNQX (10 µM) and AP-V (50 µM) at different holding potentials from −90 to −20 mV in whole-cell patch-clamp configuration with [Cl^−^]*_i _*= 10 mM in striatal MSNs from WT (black) and R6/2 (pink). ***B***, Summary of individual *E*_GABA_ recordings obtained from all IV curves with [Cl^−^]*_i _*= 10 mM in WT (*n* = 14) and R6/2 (*n* = 15; *p* = 0.0003; Student’s unpaired *t* test with Welch’s correction). ***C***, RMP with [Cl^−^]*_i _*= 10 mM from WT (*n* = 12) and R6/2 (*n* = 13; *p* = 0.0423; Student’s unpaired *t* test with Welch’s correction). ***D***, Cl^−^ driving force through GABA_A_Rs with [Cl^−^]*_i _*= 10 mM from WT (*n* = 13) and R6/2 (*n* = 13; *p* = 0.0380, Student’s unpaired *t* test). ***E***, Synaptic conductance with [Cl^−^]*_i _*= 10 mM from WT (*n* = 14) and R6/2 (*n* = 14; *p* = 0.0246, Student’s unpaired *t* test with Welch’s correction). ***F***, Similar to ***B*** but with [Cl^−^]*_i _*= 30 mM, WT (*n* = 10) and R6/2 (*n* = 19; *p* = 0.0383; Student’s unpaired *t* test). ***G***, Similar to ***C*** but with [Cl^−^]*_i _*= 30 mM, WT (*n* = 10) and R6/2 (*n* = 19; *p* = 0.0121; Student’s unpaired *t* test). ***H***, Similar to ***D*** but with [Cl^−^]*_i _*= 30 mM, WT = (*n* = 10) and R6/2 (*n* = 14; *p* = 0.0329; Student’s unpaired *t* test with Welch’s correction). ***I***, Similar to ***E*** but with [Cl^−^]*_i _*= 30 mM, WT = (*n* = 9) and R6/2 (*n* = 11; *p* = 0.0372; Student’s unpaired *t* test with Welch’s correction). ***J***, Similar to ***B*** but using gramicidin-perforated patch-clamp configuration in WT (*n* = 11) and R6/2 (*n* = 13; *p* = 0.0002; Student’s unpaired *t* test with Welch’s correction). ***K***, Similar to ***C*** but using gramicidin-perforated patch-clamp configuration in WT (*n* = 11) and R6/2 (*n* = 13; *p* = 0.0071; Student’s unpaired *t* test with Welch’s correction). ***L***, Similar to ***D*** but using gramicidin-perforated patch-clamp configuration in WT (*n* = 11) and R6/2 (*n* = 13; *p* = 0.0267; Student’s unpaired *t* test). ***M***, Similar to ***E*** but using gramicidin-perforated patch-clamp configuration in WT (*n* = 10) and R6/2 (*n* = 12; *p* = 0.0194; Student’s unpaired *t* test with Welch’s correction). ***N***, Similar to ***A*** but in MSNs from WT (black) and YAC128 (blue). ***O***, Summary of individual *E*_GABA_ recordings with [Cl^−^]*_i_* = 10 mM obtained from all IV curves in WT (*n* = 9) and YAC128 (*n* = 10; *p* < 0.0001; Student’s unpaired *t* test). ***P***, RMP with [Cl^−^]*_i_* = 10 mM from WT (*n* = 10) and YAC128 (*n* = 10; *p* = 0.0138; Student’s unpaired *t* test). ***Q***, Cl^−^ driving force through GABA_A_Rs with [Cl^−^]*_i _*= 10 mM from WT (*n* = 9) and YAC128 (*n* = 11; *p* = 0.0025, Student’s unpaired *t* test). ***R***, Synaptic conductance through GABA_A_Rs from WT (*n* = 9) and YAC128 (*n* = 10) with [Cl^−^]*_i _*= 10 mM (*p* = 0.3564; Student’s unpaired *t* test). ***S***, Similar to ***O*** but with [Cl^−^]*_i _*= 30 mM, WT (*n* = 9) and YAC128 (*n* = 8; *p* = 0.0019; Student’s unpaired *t* test). ***T***, Similar to ***P*** but with [Cl^−^]*_i _*= 30 mM, WT (*n* = 9) and YAC128 (*n* = 8; *p* = 0.0113; Student’s unpaired *t* test). ***U***, Similar to ***Q*** but with [Cl^−^]*_i _*= 30 mM, WT (*n* = 8) and YAC128 (*n* = 8; *p* = 0.0394; Student’s unpaired *t* test). ***V***, Similar to ***R*** but with [Cl^−^]*_i _*= 30 mM, WT (*n* = 9) and YAC128 (*n* = 8; *p* = 0.2139; Student’s unpaired *t* test with Welch’s correction). For all panels, the circles indicate the values from single samples, and the columns represent the mean ± SD. * *p* < 0.05, **p < 0.01 *** < 0.001. For *E*_GABA_ recordings in presymptomatic R6/2 and WT, see Extended Data Figure 1-2*F*–*N*.

10.1523/JNEUROSCI.0215-24.2024.f1-2 Figure 1-2 KCC2 expression and Cl^-^ regulation is altered in presymptomatic R6/2. (A) Representative immunoblot image for KCC2 in protein extracts from samples of striatal lysates from presymptomatic (P35-P38) WT and R6/2. O: Oligomer, M: Monomer. (B) Representative immunoblot image for NKCC1 in protein extracts from samples of striatal lysates from presymptomatic WT and R6/2. (C) Quantification of total KCC2 (oligomer + monomer) in WT (N = 7) and R6/2 (N = 6) normalized to β-actin (P = 0.0912; Student’s unpaired t*-*test). Circles indicate values from individual animals. Columns represent the mean ± SD. (D) Quantification of NKCC1 in WT (N = 7) and R6/2 (N = 6) normalized to β-Actin (P = 0.3660; Mann-Whitney test). Circles indicate values from individual animals. Whiskers indicate max to min and bar indicates the median. (E) Quantification of Oligomer to Monomer ratio of KCC2 in WT (N = 7) and R6/2 (N = 6) normalized to β-Actin (P = 0.0243; Student’s unpaired t-test). Circles indicate values from individual animals**.** Columns represent the mean ± SD**.** (F) Example IV curves of representative IPSCs (*right*) in recorded in whole-cell patch-clamp recording configuration at different holding potentials from -100  mV to -30  mV with [Cl^-^]_i_ = 10  mM from WT (*black*) and R6/2 (*pink*). (G) Summary of individual E_GABA_ recordings with [Cl^-^]_i_ = 10  mM obtained from all IV curves in WT (n = 12) and R6/2 (n = 11) (P = 0.0005; Mann-Whitney test). (H) RMP with [Cl^-^]_i_ = 10  mM from WT (n = 12) and R6/2 (n = 9) (P = 0.1889; Student’s unpaired t-test). (I) Conductance through GABA_A_R from WT (n = 12) and R6/2 (n = 11) (P = 0.1002; Mann-Whitney test). (J) Cl^-^ driving force through GABA_A_R from WT (n = 12) and R6/2 (n = 9) (P = 0.0850; Student’s unpaired t-test). (K) Similar to G but recorded in gramicidin perforated patch clamp configuration in WT (n = 9) and R6/2 (n = 9) (P = 0.0244; Mann-Whitney test). (L) Similar to H but recorded in gramicidin perforated patch clamp configuration in WT (n = 9) and R6/2 (n = 9) (P = 0.5569; Student’s unpaired t-test). (M) Similar to I but recorded in gramicidin perforated patch clamp configuration in WT (n = 9) and R6/2 (n = 9) (P = 0.4423; Student’s unpaired t-test). (N) Similar to J but recorded in gramicidin perforated patch clamp configuration in WT (n = 9) and R6/2 (n = 9) (P = 0.0550; Student’s unpaired t-test). For panels G, I, K, whiskers indicate Min to Max and bar indicates the median. For all other panels, columns represent the mean ± SD.* P < 0.05, **P < 0.01 *** < 0.001. Download Figure 1-2, TIF file.

### *E*_GABA_ is altered in striatal MSNs of symptomatic HD mice

Alterations in the expression of CCCs can change the equilibrium potential of Cl^−^ across the neuronal membrane, which in turn changes the driving force for Cl^−^ through GABA_A_Rs (*E*_GABA_), and thus the strength of synaptic inhibition. To determine whether the changes in KCC2 and NKCC1 expression observed above change *E*_GABA_, we performed whole-cell patch-clamp recordings in acute striatal slices prepared from mice at P60. *E*_GABA_ was significantly depolarized in MSNs of symptomatic R6/2 mice compared with their WT littermates (Fig. 2*A,B*; *p* = 0.0003, unpaired *t* test with Welch's correction) and was accompanied by a significant increase in the driving force for Cl^−^ through GABA_A_Rs (Fig. 2*D*; *p* = 0.0380, unpaired *t* test). These current–voltage (IV) recordings also revealed a significant increase in conductance through GABA_A_Rs in R6/2 mice (Fig. 2*E*; *p* = 0.0246, unpaired *t* test with Welch's correction). RMP was also depolarized in MSNs of symptomatic R6/2 mice (Fig. 2*C*; *p* = 0.0423, unpaired *t* test with Welch's correction).

To record *E*_GABA_ using less invasive recording techniques, we recorded *E*_GABA_ using gramicidin-perforated patch-clamp recordings. We observed a similar depolarization of *E*_GABA_ (Fig. 2*J*; *p* = 0.0002, unpaired *t* test with Welch's correction) and RMP (Fig. 2*K*; *p* = 0.0071, unpaired *t* test with Welch's correction) with similar changes in driving force for Cl^−^ (Fig. 2*L*; *p* = 0.0267, unpaired *t* test) and conductance through GABA_A_Rs (Fig. 2*M*; *p* = 0.0194, unpaired *t* test) in MSNs of symptomatic R6/2 compared with WT.

To further interrogate the relationship between CCC expression and function, we imposed a Cl^−^ load on the neuron through the patch pipette (30 mM Cl^−^), to more deeply characterize KCC2's capacity to extrude Cl^−^ (Doyon et al., 2016). Under these Cl^−^ load conditions, we recorded a significant depolarization of RMP (Fig. 2*G*; *p* = 0.0121, unpaired *t* test) and *E*_GABA_ in R6/2 MSNs compared with WT (Fig. 2*F*; *p* = 0.0383, unpaired *t* test). Cl^−^ loading also revealed similar changes in Cl^−^ driving force (Fig. 2*H*; *p* = 0.0329, unpaired *t* test with Welch's correction) and conductance (Fig. 2*I*; *p* = 0.0372, unpaired *t* test with Welch's correction). Moreover, similar results were found in the presymptomatic R6/2 (Extended Data Fig. 2-2*F*–*N*) and symptomatic YAC128 mouse model (Fig. 2*N–V*). Symptomatic YAC128 mice displayed a depolarization of *E*_GABA_ under low (Fig. 2*N, O*; *p* < 0.0001, unpaired *t* test) and high Cl^−^ conditions (Fig. 2*S*; *p* = 0.0019, unpaired *t* test). RMP was also depolarized (Fig. 2*P,T*), and the driving force for GABA was altered (Fig. 2*Q,U*), with no change in the GABA_A_R conductance (Fig. 2*R,V*). Taken together, these data demonstrate that Cl^−^ homeostasis may be disrupted in symptomatic HD mice.

### Cl^−^ regulation is selectively impaired in D2 MSNs of R6/2 mice

**Figure 3.**
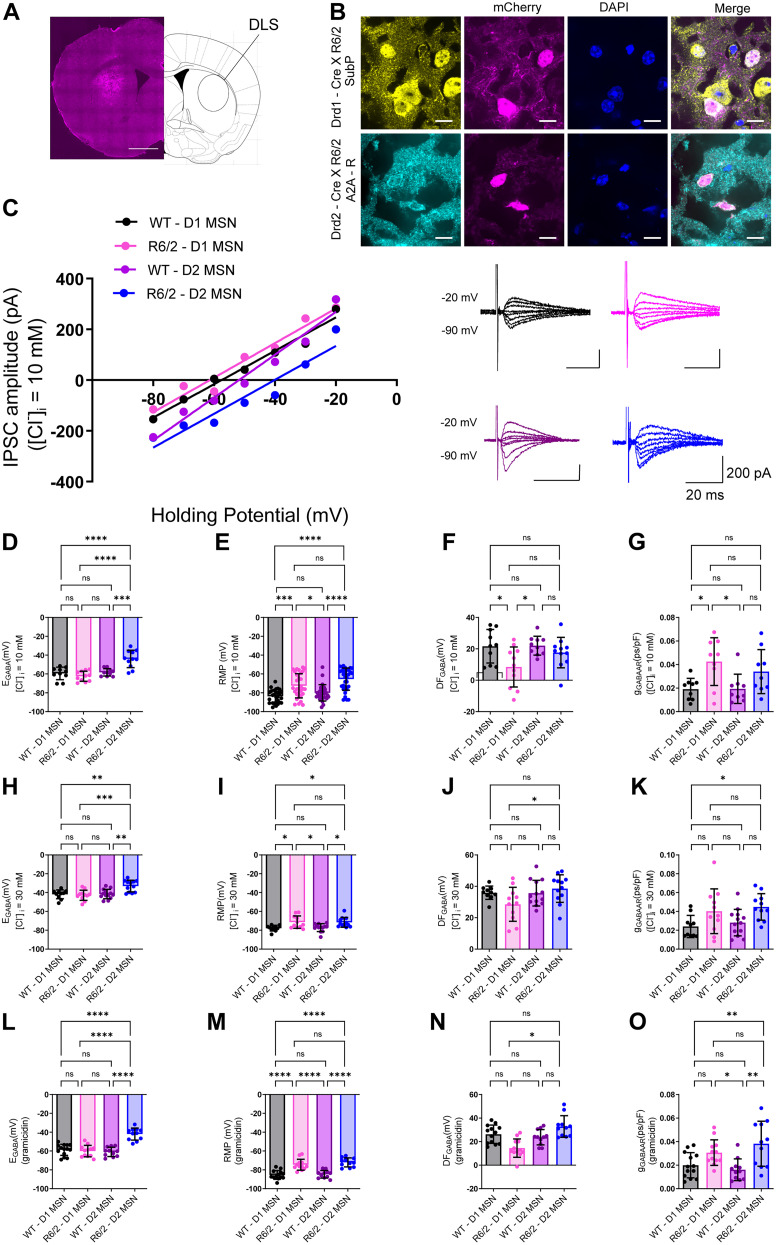
KCC2 function is altered specifically in D2 MSNs of R6/2 mice. ***A***, Image showing infection site of AAV5-mCherry in the dorsolateral striatum. ***B***, Top, Representative images of substance P expression in Drd1-Cre x R6/2 mice injected with AAV5-mCherry. ***B***, Bottom, Representative images of A2A-receptor expression in Drd2-Cre x R6/2 mice injected with virus containing mCherry. Scale bar, 10 µm. ***C***, Example IV curves of representative IPSCs (right) induced by electrical stimulation in the presence of glutamate blockers CNQX (10 µM) and AP-V (50 µM) at different holding potentials from −90 to −20 mV in whole-cell patch-clamp configuration with [Cl^−^]*_i_* = 10 mM in WT D1 MSNs (black), R6/2 D1 MSNs (pink), WT D2 MSNs (purple), and R6/2 D2 MSNs (blue)*. **D***, Summary of individual *E*_GABA_ recordings obtained from all IV curves in WT D1 MSNs (*n* = 10), WT D2 MSNs (*n* = 10), R6/2 D1 MSNs (*n* = 11), and R6/2 D2 -MSNs (*n* = 11) with [Cl^−^]*_i_* = 10 mM (*p* = 0.0002; two-way analysis of ANOVA followed by Sidak’s multiple-comparisons test, *F*_(1,38) _= 17.57). ***E***, RMP from WT D1 MSNs (*n* = 28), WT D2 MSNs (*n* = 28), R6/2 D1 MSNs (*n* = 30), and R6/2 D2 MSNs (*n* = 31) with [Cl^−^]*_i_* = 10 mM (*p* = 0.4381; two-way analysis of ANOVA followed by Sidak’s multiple-comparisons test, *F*_(1,112) _= 0.6057). ***F***, Cl^−^ driving force through GABA_A_Rs from WT D1 MSNs (*n* = 10), WT D2 MSNs (*n* = 10), R6/2 D1 MSNs (*n* = 11), and R6/2 D2 MSNs (*n* = 11) with [Cl^−^]*_i _*= 10 mM (*p* = 0.1674; two-way analysis of ANOVA followed by Sidak’s multiple-comparisons test, *F*_(1,38) _= 1.981). ***G***, Synaptic conductance from WT D1 MSNs (*n* = 9), WT D2 MSNs (*n* = 9), R6/2 D1 MSNs (*n* = 9), and R6/2 D2 MSNs (*n* = 9) with [Cl^−^]*_i _*= 10 mM (*p* = 0.4185; two-way analysis of ANOVA followed by Sidak’s multiple-comparisons test, *F*_(1,32) _= 0.6717). ***H***, Similar to ***D*** but with [Cl^−^]*_i_* = 30 mM, WT D1 MSNs (*n* = 10), WT D2 MSNs (*n* = 13), R6/2 D1 MSNs (*n* = 11), and R6/2 D2 MSNs (*n* = 12; *p* = 0.0048; two-way analysis of ANOVA followed by Sidak’s multiple-comparisons test, *F*_(1,42) _= 8.868). ***I***, Similar to ***E*** but with [Cl^−^]*_i _*= 30 mM, WT D1 MSNs (*n* = 10), WT D2 MSNs (*n* = 13), R6/2 D1 MSNs (*n* = 11), and R6/2 D2 MSNs (*n* = 12; *p* = 0.7806; two-way analysis of ANOVA followed by Sidak’s multiple-comparisons test, *F*_(1,42) _= 0.07861). ***J***, Similar to ***F*** but with [Cl^−^]*_i_* = 30 mM, WT D1 MSNs (*n* = 10), WT D2 MSNs (*n* = 13), R6/2 D1 MSNs (*n* = 11), and R6/2 D2 MSNs (*n* = 12; *p* = 0.0459; two-way analysis of ANOVA followed by Sidak’s multiple-comparisons test, *F*_(1,42) _= 4.234). ***K***, Similar to ***G*** but with [Cl^−^]*_i _*= 30 mM, WT D1 MSNs (*n* = 10), WT D2 MSNs (*n* = 13), R6/2 D1 MSNs (*n* = 11), and R6/2 D2 MSNs (*n* = 11; *p* = 0.9652; two-way analysis of ANOVA followed by Sidak’s multiple-comparisons test, *F*_(1,41) _= 0.001923). ***L***, Similar to ***D*** but using gramicidin-perforated patch-clamp configuration, WT D1 MSNs (*n* = 14), WT D2 MSNs (*n* = 11), R6/2 D1 MSNs (*n* = 12), and R6/2 D2 MSNs (*n* = 11; *p* < 0.0001; two-way analysis of ANOVA followed by Sidak’s multiple-comparisons test, *F*_(1,44) _= 35.29). ***M***, Similar to ***E*** but using gramicidin-perforated patch-clamp configuration, WT D1 MSNs (*n* = 13), WT D2 MSNs (*n* = 11), R6/2 D1 MSNs (*n* = 12), and R6/2 D2 MSNs (*n* = 11; *p* = 0.5458; two-way analysis of ANOVA followed by Sidak’s multiple-comparisons test, *F*_(1,43) _= 0.3708). ***N***, Similar to ***F*** but using gramicidin-perforated patch-clamp configuration, WT D1 MSNs (*n* = 13), WT D2 MSNs (*n* = 11), R6/2 D1 MSNs (*n* = 12), and R6/2 D2 MSNs (*n* = 11; *p* = 0.0077; two-way analysis of ANOVA followed by Sidak’s multiple-comparisons test, *F*_(1,43) _= 7.821). ***O***, Similar to ***G*** but using gramicidin-perforated patch-clamp configuration, WT D1 MSNs (*n* = 13), WT D2 MSNs (*n* = 11), R6/2 D1 MSNs (*n* = 12), and R6/2 D2 MSNs (*n* = 11; *p* = 0.1386; two-way analysis of ANOVA followed by Sidak’s multiple-comparisons test, *F*_(1,43) _= 2.277). For all panels, the circles indicate the values from single samples, and all columns represent mean ± SD. *p < 0.05, **p < 0.01 ***<0.001. For a comparison of passive membrane properties of D1 and D2 MSNs in R6/2 and WT mice, see Extended Data Table 3-1.

While *E*_GABA_ from R6/2 MSNs was more depolarized, some cells displayed comparable *E*_GABA_ values to WT mice (Fig. 2*B*). This variability led us to ask whether there are differences in synaptic dysfunction between D1 and D2 MSNs during the early symptomatic phase of HD. To answer this question, *E*_GABA_ was recorded from fluorescently labeled D1 and D2 MSNs in WT and R6/2 mice during the early stages of the symptomatic phase (P55–P65) to detect any differences in Cl^−^ homeostasis. There were no differences in intrinsic membrane properties between MSN subtypes of either WT or R6/2 mice; however, there were significant differences between genotypes (Extended Data Table 3-1). As expected, D2 MSNs in R6/2 mice displayed a significantly depolarized *E*_GABA_ compared with D2 MSNs in WT mice under low (Fig. 3*C,D*; *F*_(1,38)_ = 17.57, *p* = 0.0002, two-way ANOVA followed by Sidak's multiple-comparisons test)**,** high levels of Cl^−^ in the recording pipette (Fig. 3*E*; *F*_(1,42)_ = 8.868, *p* = 0.0048, two-way ANOVA followed by Sidak's multiple-comparisons test) and with perforated patch-clamp recordings (Fig. 3*L*; *F*_(1,44)_ = 35.29, *p* < 0.0001, two-way ANOVA followed by Sidak's multiple-comparisons test)**.** Interestingly, in R6/2 mice, *E*_GABA_ in D2 MSNs was significantly depolarized compared with D1 MSNs under all recording conditions (Fig. 3*D,E,L*). Moreover, a change driving force of Cl^−^ through GABA_A_Rs was observed in R6/2 D1 MSNs compared with R6/2 D2 MSNs using high Cl^−^ and gramicidin-containing ICS (Fig. 3*J,N*), although this difference was not detected under low Cl^−^ conditions (Fig. 3*F*; *F*_(1,38)_ = 1.981, *p* = 0.1674, two-way ANOVA followed by Sidak's multiple-comparisons test). Similar to our previous observations, there were differences in Cl^−^ conductance through GABA_A_Rs between genotypes but not between cell types in either WT or R6/2 (Fig. 3*G,K,O*). Taken together, the early Cl^−^ dysfunction specifically in D2 MSNs may contribute to the enhanced susceptibility of D2 MSNs to neurodegeneration in HD.

10.1523/JNEUROSCI.0215-24.2024.t3-1 Table 3-1 Membrane properties of D1 and D2 neurons from R6/2 and WT mice. All tests were subjected to a two-way ANOVA with Tukey’s Multiple comparisons test. Values show Mean ± SEM. Download Table 3-1, XLS file.

### Cl^−^ regulation is altered in the GPe of symptomatic R6/2 mice

**Figure 4.**
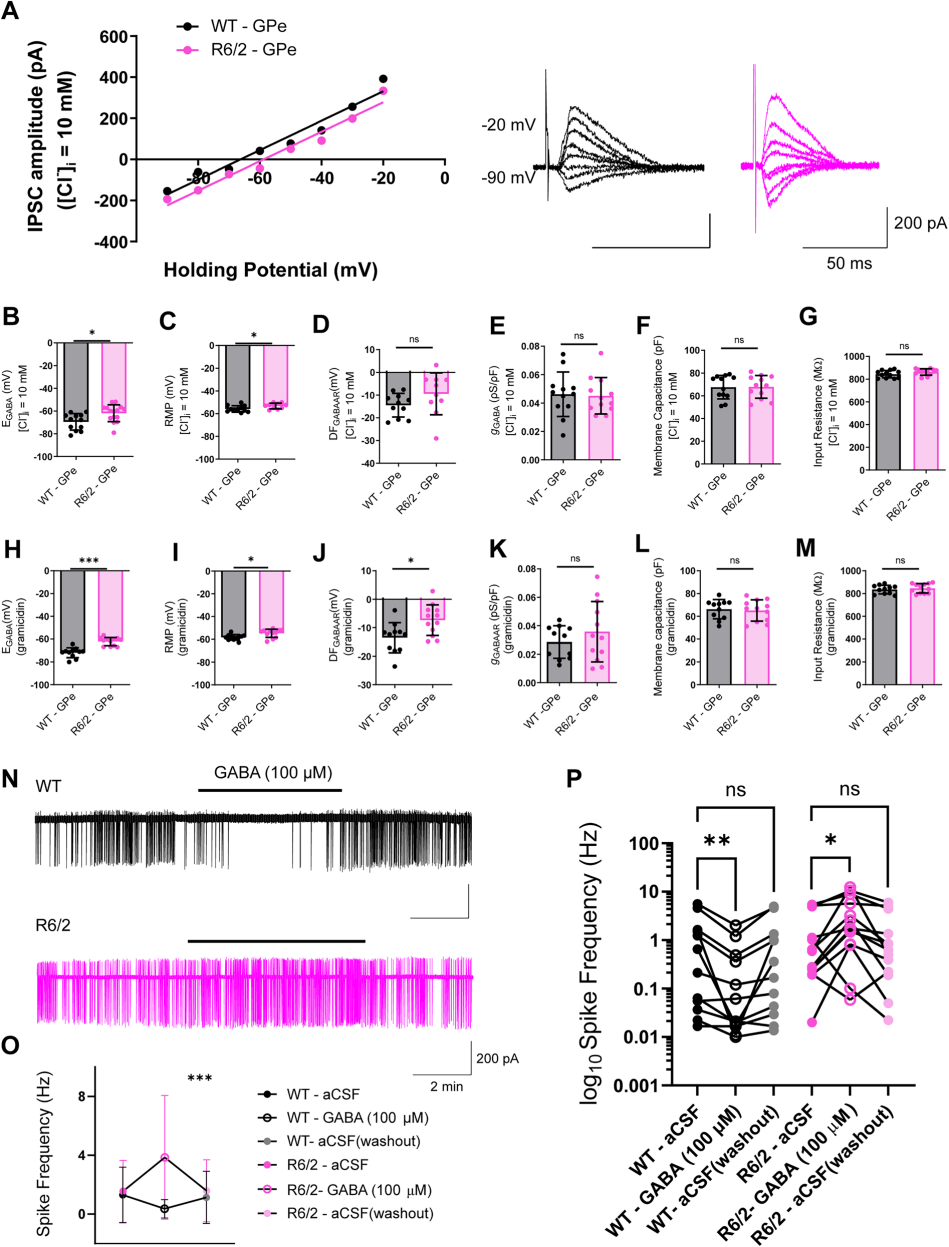
Cl^−^ regulation is altered in GPe neurons of R6/2 mice. ***A***, Example IV curves of representative IPSCs (right) induced by electrical stimulation in the presence of glutamate blockers CNQX (10 µM) and AP-V (50 µM) at different holding potentials from −90 to −20 mV in whole-cell patch-clamp configuration with [Cl^−^]*_i_* = 10 mM in GPe neurons from WT (black) and R6/2 (pink). ***B***, Summary of individual *E*_GABA_ recordings with [Cl^−^]*_i _*= 10 mM obtained from all IV curves in WT (*n* = 12) and R6/2 (*n* = 15; *p* = 0.0130; Student’s unpaired *t* test). ***C***, RMP with [Cl^−^]*_i _*= 10 mM from WT (*n* = 11) and R6/2 (*n* = 10; *p* = 0.0392; unpaired Student’s *t* test). ***D***, Cl^−^ driving force through GABA_A_Rs with [Cl^−^]*_i _*= 10 mM from WT (*n* = 11) and R6/2 (*n* = 10; *p* = 0.1363; Student’s unpaired *t* test). ***E***, Conductance through GABA_A_Rs with [Cl^−^]*_i _*= 10 mM from WT (*n* = 12) and R6/2 (*n* = 12; *p* = 0.8489; Student’s unpaired *t* test). ***F***, Membrane capacitance with [Cl^−^]*_i _*= 10 mM from WT (*n* = 12) and R6/2 (*n* = 12; *p* = 0.9605; Student’s unpaired *t* test). ***G***, Input resistance with [Cl^−^]*_i _*= 10 mM from WT (*n* = 12) and R6/2 (*n* = 12; *p* = 0.1313; unpaired Student’s *t* test). ***H***, Similar to ***B*** but using gramicidin-perforated patch-clamp configuration from WT (*n* = 11) and R6/2 (*n* = 12; *p* < 0.0001; Student’s unpaired *t* test). ***I***, Similar to ***C*** but using gramicidin-perforated patch-clamp configuration from WT (*n* = 11) and R6/2 (*n* = 12; *p* = 0.0111; Student’s unpaired *t* test). ***J***, Similar to ***D*** but using gramicidin-perforated patch-clamp configuration from WT (*n* = 11) and R6/2 (*n* = 12; *p* = 0.0118; Student’s unpaired *t* test). ***K***, Similar to ***E*** but using gramicidin-perforated patch-clamp configuration from WT (*n* = 11) and R6/2 (*n* = 12; *p* = 0.3242; Student’s unpaired *t* test). ***L***, Similar to ***F*** but using gramicidin-perforated patch-clamp configuration from WT (*n* = 11) and R6/2 (*n* = 12; *p* = 0.7690; Student’s unpaired *t* test). ***M***, Similar to ***G*** but using gramicidin-perforated patch-clamp configuration from WT (*n* = 11) and R6/2 (*n* = 12; *p* = 0.5080; Student’s unpaired *t* test). ***N***, Sample traces of spontaneous spiking activity in cell-attached patch-clamp configuration in GPe neurons from WT (black) and R6/2 (pink) at baseline (aCSF), during bath application of GABA (100 µM) and during washout period. ***O***, Quantification of mean spike frequency for WT (*n* = 12) and R6/2 (*n* = 13) at baseline, after bath application of GABA (100 µM), and during washout (WT GABA vs R6/2 GABA, *p* = 0.0006, Mann–Whitney test). The circles indicate mean ± SD. ***P***, Quantification of log_10_ spike frequency for WT (*n* = 12; black) at baseline, after bath application of GABA (100 µM), and during washout period (*p* = 0.0112; repeated-measures one-way ANOVA followed by Dunnett’s multiple-comparisons test, *F*_(2,22) _= 5.553); R6/2 (*n* = 13; pink) at baseline, after bath application of GABA (100 µM), and during washout period (*p* = 0.0072; repeated-measures one-way ANOVA followed by Dunnett’s multiple-comparisons test, *F*_(2,24) _= 6.111). For all other panels, the circles indicate the values from single samples, and all columns represent mean ± SD. * *p* < 0.05, **p < 0.01, *** < 0.001. For recordings in SNr neurons in R6/2 and WT, see Extended Data Figure 4-3.

In GPe neurons of symptomatic HD mice, GABA-mediated inhibition is altered and has been linked to impaired astrocytic GABA clearance in the synaptic cleft (Barry et al., 2020) as well as changes in GABA_A_R subunits (Du et al., 2017; Perez-Rosello et al., 2019). Based on the known changes in GABAergic inhibition in GPe neurons, we investigated whether Cl^−^ dysfunction is also present in GPe neurons of symptomatic R6/2 mice. To test this, we recorded *E*_GABA_ in GPe neurons of WT and R6/2 mice. In GPe neurons of symptomatic R6/2 mice, both *E*_GABA_ (Fig. 4*A,B*; *p* = 0.0130, unpaired *t* test) and RMP were depolarized (Fig. 4*C*; *p* = 0.0392, unpaired *t* test) compared with age-matched WT in low Cl^−^ conditions and using gramicidin-containing ICS (Fig. 4*H,I*). There was no change in the driving force for GABA (Fig. 4*D*; *p* = 0.1363, unpaired *t* test) using low Cl^−^ ICS; however, alterations in driving force were observed with gramicidin-containing ICS (Fig. 4*J*). There were no changes in GABA_A_R conductance (Fig. 4*E,K*), and passive membrane properties were unchanged in R6/2 mice compared with WT, specifically membrane capacitance (Fig. 4*F,L*) and input resistance (Fig. 4*G,M*). In the GPe, *E*_GABA_ sits close to RMP, which means that a change in either parameter can lead to a change in GABA polarity. To determine whether the depolarizations in *E*_GABA_ and RMP that we recorded resulted in a change in GABA polarity, we bath applied GABA while recording the firing frequency of GPe neurons. We found that GABA led to increased firing frequency in GPe neurons of R6/2 mice, while producing the opposite effect in the WT (Fig. 4*N–P*). In contrast, we found that *E*_GABA_ (Extended Data Fig. 4-3*A,B,H*) and RMP (Extended Data Fig. 4-3*C,I*) were unchanged in the SNr of R6/2 compared with WT mice. Additionally, GABA signaling appeared to remain intact (Extended Data Fig. 4-3*N–P*) including the driving force for GABA (Extended Data Fig. 4-3*D–J*) and conductance through GABA_A_Rs (Extended Data Fig. 4-3*E,K*). Moreover, passive membrane properties of SNr neurons were unaltered in R6/2 mice (Extended Data Fig. 4-3*F,G,L,M*). These findings demonstrate that while Cl^−^ regulation appears unaffected in SNr neurons of early symptomatic R6/2 mice, Cl^−^ handling is impaired in R6/2 GPe neurons.

10.1523/JNEUROSCI.0215-24.2024.f4-3 Figure 4-3 Cl^-^ regulation is unaltered in the SNr of R6/2 mice. (A) Example IV curves of representative IPSCs (*right*) induced by electrical stimulation in the presence of glutamate blockers CNQX (10 μM) and AP-V (50 μM) at different holding potentials from -100 to -30  mV in whole-cell patch-clamp configuration with [Cl^-^]_i_ = 10  mM in SNr neurons from WT (*black*) and R6/2 (*pink*). (B) Summary of individual E_GABA_ recordings with [Cl^-^]_i_ = 10  mM obtained from all IV curves in WT (n = 11) and R6/2 (n = 12) (P = 0.4015; Student’s unpaired t-test). (C) RMP with [Cl^-^]_i_ = 10  mM from WT (n = 11) and R6/2 (n = 11) (P = 0.5676; Student’s unpaired t-test)**.** (D) Cl^-^ driving force through GABA_A_Rs with [Cl^-^]_i_ = 10  mM from WT (n = 11) and R6/2 (n = 11) (P = 0.3248; Unpaired student’s t-test). (E) Conductance through GABA_A_Rs with [Cl^-^]_i_ = 10  mM from WT (n = 10) and R6/2 (n = 11) (P = 0.1517; Mann-Whitney test). Whiskers indicate Min to Max and bar indicates median. (F) Membrane capacitance with [Cl^-^]_i_ = 10  mM from WT (n = 11) and R6/2 (n = 11) (P = 0.2539; Student’s unpaired t-test). (G) Input resistance with [Cl^-^]_i_ = 10  mM from WT (n = 11) and R6/2 (n = 11) (P = 0.0958; Student’s unpaired t-test). (H) Similar to B but recorded in gramicidin perforated patch clamp configuration from WT (n = 10) and R6/2 (n = 10) (P = 0.5374; Student’s unpaired t-test)**.** (I) Similar to C but recorded in gramicidin perforated patch clamp configuration from WT (n = 10) and R6/2 (n = 10) (P = 0.5374; Student’s unpaired t-test)**.** (J) Similar to D but recorded in gramicidin perforated patch clamp configuration from WT (n = 10) and R6/2 (n = 10) (P = 0.6506; Student’s unpaired t-test)**.** (K) Similar to E but recorded in gramicidin perforated patch clamp configuration from WT (n = 10) and R6/2 (n = 10) (P = 0.7929; Student’s unpaired t-test)**.** (L) Similar to F but recorded in gramicidin perforated patch clamp configuration from WT (n = 10) and R6/2 (n = 10) (P = 0.7666; Student’s unpaired t-test)**.** (M) Similar to G but recorded in gramicidin perforated patch clamp configuration from WT (n = 10) and R6/2 (n = 10) (P = 0.6517; Student’s unpaired t-test)**.** (N) Sample traces of spontaneous spiking activity in cell-attached patch clamp configuration in SNr neurons from WT (*black) and* R6/2 *(pink)* at baseline, during bath application of GABA (100 μM) and during wash out period. (O) Quantification of mean spike frequency for WT (n = 14) and R6/2 (n = 9) at baseline, after bath application of GABA (100 μM) and during washout (WT – GABA vs. R6/2 – GABA, P = 0.4295, Mann-Whitney test). Circles represent the mean ± SD. (P) Quantification of log_10_ spike frequency for WT (n = 14) (*black*) at baseline, after bath application of GABA (100 μM), and during washout period (P = 0.0017; Repeated measures One-way ANOVA followed by Dunnett’s multiple comparisons test F_(2,26)_ = 8.217); R6/2 (n = 9) (*pink*) at baseline, after bath application of GABA (100 μM), and during washout period (P = 0.0011; Repeated measures One-way ANOVA followed by Dunnett’s multiple comparisons test F_(2,16)_ = 10.75). Circles indicate single samples and columns represent the mean. For all panels (except E and O), columns represent the mean ± SD * P < 0.05, **P < 0.01 *** < 0.001. Download Figure 4-3, TIF file.

### Bumetanide treatment delays the onset of motor deficits in R6/2 mice

**Figure 5.**
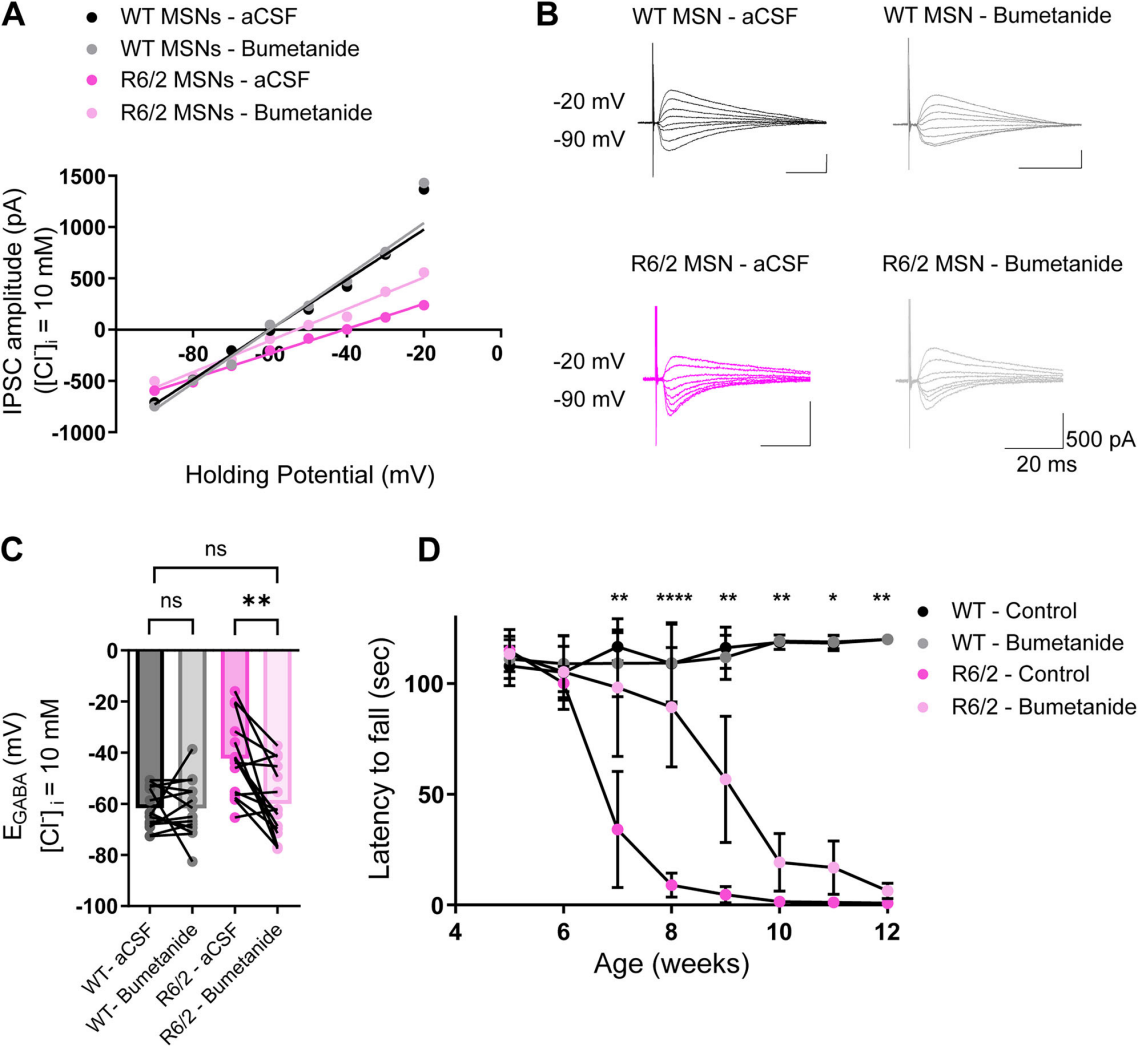
Bumetanide restores *E*_GABA_ and delays the onset of motor impairments. ***A***, Example IV curves of representative IPSCs recorded in whole-cell patch-clamp recording configuration at different holding potentials from −90 mV to −20 mV with [Cl^−^]*_i _*= 10 mM. ***B***, Representative IPSC traces from WT mice before (black) and after perfusion with bumetanide (10 µM; dark gray) and R6/2 mice before (pink) and after perfusion with bumetanide (10 µM; light gray). ***C***, Summary of *E*_GABA_ before and after application of bumetanide in WT (*n* = 14; *p* = 0.9679; Student’s paired *t* test); R6/2 before and after application of bumetanide (*n* = 15; *p* = 0.0016; Student’s paired *t* test); and *E*_GABA_ before bumetanide in WT (*n* = 14) and after bumetanide in R6/2 (*n* = 15; *p* = 0.6994; Student’s unpaired *t* test). The circles indicate the values from single samples, and the columns represent the mean. ***D***, Rotarod performance, Mice were trained for 1 week and tested where they were placed on a rotarod at a constant rate of 10 rpm for 2 min. WT control (*N* = 13), WT bumetanide (*N* = 12), R6/2 control (*N* = 7), R6/2 bumetanide (*N* = 10). The asterisks are shown only for R6/2 control and R6/2 bumetanide (*p* < 0.0001; two-way ANOVA with Tukey’s post hoc multiple-comparisons test, *F*_(21,266) _= 68.40). For statistical details, see Extended Data Table 5-2. The circles indicate mean ± SD. *p < 0.05, **p < 0.01, *** < 0.001.

Bumetanide is an FDA-approved loop diuretic widely used in many neurological diseases associated with pathological increases in intracellular Cl^−^ (Puskarjov et al., 2014; Ben-Ari, 2017). Bumetanide blocks NKCC1 activity to restore physiological intracellular Cl^−^ levels and GABAergic inhibition. To determine the effects of bumetanide on *E*_GABA_ in vitro, we measured *E*_GABA_ from MSNs of R6/2 and WT mice before and after bath perfusion of bumetanide. Bath application of bumetanide effectively hyperpolarized *E*_GABA_ in R6/2 MSNs (Fig. 5*A–C*, *p* = 0.0016, paired *t* test), and as expected, bumetanide had no effect on WT *E*_GABA_ values (Fig. 5*A–C*, *p* = 0.9679, paired *t* test), since [Cl^−^]*_i_* is predominantly regulated by KCC2 in mature neurons (Wang et al., 2002). Importantly, bath application of bumetanide hyperpolarized *E*_GABA_ in R6/2 MSNs similar to WT levels (Fig. 5*A,B*, *p* = 0.6994, unpaired *t* test). We then tested the effects of inhibiting NKCC1 in vivo with daily IP injections of bumetanide and assessed motor coordination using the rotarod. Daily bumetanide treatment delayed motor deficits in R6/2 mice compared with controls (Fig. 5*C*, Extended Data Table 5-2) and had no effect on WT Rotarod performance. Altogether, this demonstrates that CCC dysfunction may contribute to the behavioral impairments associated with HD.

10.1523/JNEUROSCI.0215-24.2024.t5-2 Table 5-2 Chronic IP injection of bumetanide delays the onset of motor deficits in R6/2 mice. All tests were subjected to a two-way ANOVA with Tukey Multiple comparisons test. The difference indicates the difference between the mean latency to fall (s) on the Rotarod for each group. Download Table 5-2, XLS file.

### Overexpression of KCC2 in D2 MSNs improves motor function in R6/2 mice

**Figure 6.**
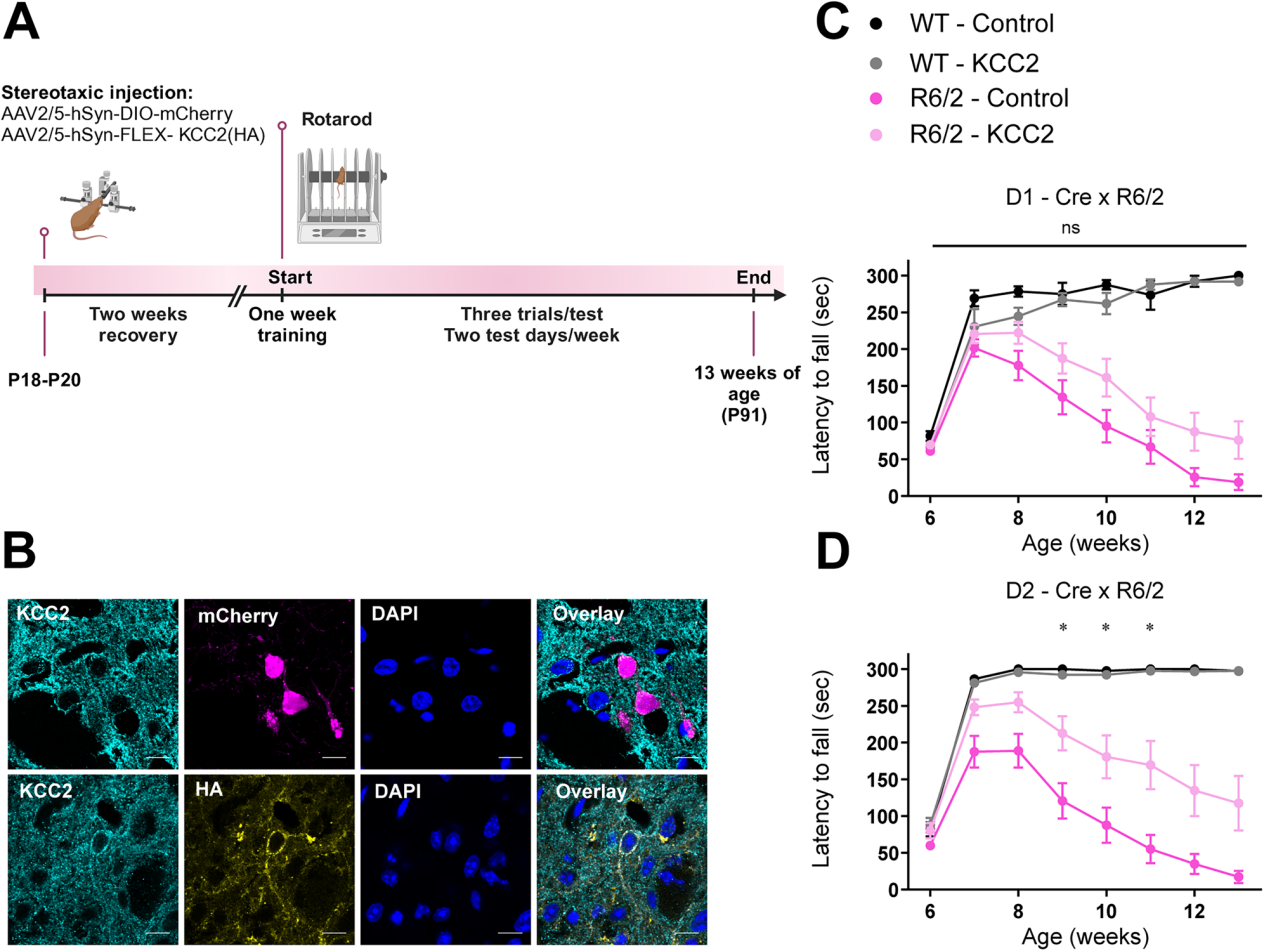
Overexpressing KCC2 in D2 MSNs improves motor behavior in R6/2 mice. ***A***, Experimental timeline, Mice were injected with either AAV-mCherry or AAV-KCC2 at P18–P20. After 2 weeks, mice were trained for 1 week on the rotarod that accelerated quickly from 0 to 5 rpm and then gradually from 5 to 20 rpm. Mice were tested at a constant rate of 10 rpm for 300 s. Mice were tested for 2 d per week with three trials per test until mice were 13 weeks of age. ***B***, Sample image from a D2-Cre x R6/2 mouse injected with AAV2/5-hSyn-DIO-mCherry (top) and a D2-Cre x R6/2 mouse injected with AAV2/5-hSyn-FLEX-KCC2(HA) (bottom). Scale bar, 10 µm. ***C***, Rotarod performance of D1-Cre x R6/2 mice injected with either AAV-mCherry or AAV-KCC2; WT injected with AAV-KCC2 (*N* = 13), WT injected with AAV-mCherry (*N* = 11), R6/2 injected with AAV-KCC2 (*N* = 20), R6/2 injected with AAV-mCherry (*N* = 14). *p* < 0.0001; two-way ANOVA with Tukey’s post hoc multiple-comparisons test, *F*_(21,378) _= 15.43. For statistical details, see Extended Data Table 6-3. ***D***, Rotarod performance of D2-Cre x R6/2 mice injected with either AAV-mCherry or AAV-KCC2; WT injected with AAV-KCC2 (*N* = 16), WT injected with AAV-mCherry (*N* = 14), R6/2 injected with AAV-KCC2 (*N* = 13), R6/2 injected with AAV-mCherry (*N* = 15). *p* < 0.0001; two-way ANOVA with Tukey’s post hoc multiple-comparisons test, *F*_(21,378) _= 13.50. For statistical details, see Extended Data Table 6-4. The asterisks are shown only for R6/2 injected with AAV-mCherry versus R6/2 injected with AAV-KCC2. The circles indicate mean ± SEM.

Although bumetanide is widely used to reduce pathological increases in Cl^−^ levels and attenuates many disorders in experimental conditions (Kahle et al., 2008; Pressey et al., 2023), its effectiveness may be limited due to its target specificity and blood–brain penetration (Römermann et al., 2017; Löscher and Kaila 2022). KCC2 is emerging as an appealing target, as recently suggested (Andrews et al., 2020; Serranilla and Woodin 2021), due in large part to its neuronal specificity. Given that Cl^−^ dysfunction was D2 MSN-specific, we assessed the effects of KCC2 overexpression specifically in either D1 MSNs or D2 MSNs on motor function (Fig. 6*A*). At age P18–P20, WT and R6/2 mice received either a virus containing KCC2-HA or mCherry as a control (Fig. 6*A,B*). KCC2 overexpression in D2 MSNs of R6/2 mice improved motor function in mice at 9–11 weeks of age (Fig. 6*D*, Extended Data Table 6-4). When KCC2 was overexpressed in D1 MSNs of R6/2 mice, performance on the rotarod appeared to improve generally (Weeks 12–13), although this did not reach statistical significance (Fig. 6*C*, Extended Data Table 6-3). Altogether, these findings suggest that CCCs play an important role in preserving motor function during the early stages of the disease.

10.1523/JNEUROSCI.0215-24.2024.t6-3 Table 6-3 Overexpression of KCC2 in D1 MSNs does not impact motor behaviour of R6/2 mice. Table shows there are no differences in rotarod performance when a AAV5-hSyn-KCC2(HA) is expressed in D1 MSNs of symptomatic R6/2 mice compared to R6/2 mice who received AAV5-hSyn -DIO-mCherry (control). All tests were subjected to a two-way ANOVA with Tukey’s Multiple comparisons test. The difference indicates the difference between the mean latency to fall (s) on the Rotarod for each group. Download Table 6-3, XLS file.

10.1523/JNEUROSCI.0215-24.2024.t6-4 Table 6-4 Overexpression of KCC2 in D2 MSNs improves motor behaviour of R6/2 mice. Table shows there are differences in rotarod performance when a AAV5-hSyn-KCC2(HA) is expressed in D2 MSNs of symptomatic R6/2 mice compared to R6/2 mice who received AAV5-hSyn -DIO-mCherry (control). All tests were subjected to a two-way ANOVA with Tukey’s Multiple comparisons test. The difference indicates the difference between the mean latency to fall (s) on the Rotarod for each group. Download Table 6-4, XLS file.

## Discussion

Despite the known impairments of CCCs in HD, the role of CCCs in the direct and indirect pathways of the basal ganglia has not been fully characterized in the early symptomatic phase of this disease. We found that Cl^−^ regulaton was altered in D2 MSNs and in GPe neurons contributing to altered GABA signaling in R6/2 within the indirect pathway. By overexpressing KCC2 in D2 MSNs, we delayed the onset of motor impairments and, in doing so, revealed that Cl^−^ regulaton may be directly or indirectly involved in the enhanced susceptibility of D2 MSNs to neurodegeneration during HD progression.

### KCC2 expression is decreased and Cl^−^ regulation is impaired in symptomatic MSNs of R6/2 mice

Our primary objective was to determine whether Cl^−^ homeostasis is differentially altered in D1 and D2 MSNs and their output structures, and whether this altered expression contributes to the pattern of degeneration between these two principal striatal cell types. This rationale was based in large part on the previous literature implicating CCCs in HD (Hsu et al., 2017; Dargaei et al., 2018; Hsu et al., 2019). More specifically, in the striatum of R6/2 mice, NKCC1 is increased at late stages of the disease (10.5-week-old mice; Hsu et al., 2019), while KCC2 and its positive regulator, brain-type creatine kinase B, are decreased in 12-week-old R6/2 mice (Hsu et al., 2017). Given that declines in KCC2 often accompany elevations in NKCC1 leading to synaptic dysfunction (Kahle et al., 2008), we chose to examine KCC2 at an earlier symptomatic phase (8.5 weeks). As we had hypothesized, we found a decrease in KCC2 and an increase in NKCC1 protein expression in the striatum, which is consistent with our previous observation in the hippocampus (Dargaei et al., 2018). In both the present study and the previous reports (Hsu et al., 2017; Hsu et al., 2019), the decreases in KCC2 and increases in NKCC1 translated, as predicted, to a depolarization of *E*_GABA_ that reduced the driving force for Cl^−^ through GABA_A_Rs.

We also observed increased conductance through GABA_A_Rs in MSNs of symptomatic R6/2 mice (Fig. 2*E,I,M*), which was absent in YAC128 MSNs (Fig. 2*R,V*). This likely reflects differences in mHTT pathogenic mechanisms, as the R6/2 model represents juvenile-onset HD (Mangiarini et al., 1996), while the YAC128 model, with slower disease progression, reflects typical late-onset HD (Slow et al., 2003). Although both models show dysregulated Cl^−^, suggesting the importance of CCCs in synaptic dysfunction in HD, these differences emphasize the need for multiple mouse models.

### *E*_GABA_ is depolarized in D2 MSNs in the early symptomatic phase

With the discovery that KCC2 and NKCC1 protein expression and function are disrupted in the striatum of early symptomatic HD, we next asked whether there is a differential impact on D1 or D2 MSNs. This is a critically important question in HD research given that D2 MSNs demonstrate earlier synaptic dysfunction and degeneration compared with their D1 counterparts (Reiner et al., 1988; Albin et al., 1992), a question which had not been explored in the previous studies on CCCs in HD. We found that D2 MSNs were affected more profoundly, as demonstrated by their more depolarized *E*_GABA_ values, compared with D1 MSNs.

GABA signaling is increased on to the indirect (D2) pathway in the striatum of HD mouse models (Cepeda et al., 2013, 2004), which is consistent with our observation of increased GABAergic conductance. Normally, an increase in GABAergic conductance translates into stronger inhibition; however, when increased conductance is coupled with a depolarization of *E*_GABA_, the net result is often a weakening of inhibition, beyond that produced by the depolarization of *E*_GABA_ alone. It is possible that the decrease in KCC2 and the increase in NKCC1 are a homeostatic neuroprotective response to counteract the increased GABA conductance, despite the potentially deleterious effect of facilitating increased excitability and potentially excitotoxicity. However, an alternate explanation stems from the differential connectivity and excitability of the indirect and direct pathways. D2 MSNs receive preferential innervation from pyramidal tract–type cortical neurons that are believed to release more glutamate onto D2 MSNs, compared with D1 s (Francelle et al., 2014). D2 MSNs are also more excitable than D1 MSNs (Cepeda et al., 2007). Taken together, these neuroanatomical and electrophysiological differences could contribute to D2 MSN vulnerability to excitotoxicity (Lei et al., 2004; Francelle et al., 2014). If we now consider that upon D2 MSN neurodegeneration in HD, cortical inputs to these striatal neurons regress (Cepeda et al., 2003, 2007), homeostatic plasticity may result in altered Cl^−^ regulation in D2 MSNs to counteract the loss of excitatory input.

In addition to the two homeostatic plasticity-based hypotheses presented above to explain the altered Cl^−^ regulation in D2 MSNs in the early symptomatic phase of HD, a third mechanistic explanation stems from impaired BDNF-TrkB signaling in this disease. D2 MSNs exhibit higher TrkB levels compared with D1 MSNs (Baydyuk and Xu 2014). The well-known alterations in BDNF-TrkB signaling in HD (Plotkin et al., 2014), combined with the regulatory effects of BDNF-TrkB signaling on KCC2 (Lee-Hotta et al., 2019; Rivera et al., 2002), open the possibility that impaired BDNF-TrkB signaling leads to altered Cl^−^ homeostasis preferentially in D2 MSNs, playing a causal role in their enhanced susceptibility in HD.

### GABA is excitatory in the GPe

Downstream targets of D1 and D2 MSNs, the SNr and Gpe, respectively, are also differentially affected in HD mouse models leading to imbalanced BG output (Barry et al., 2018). When recording from GPe neurons, we found that both *E*_GABA_ and RMP were depolarized in R6/2 neurons in either recording condition (whole cell or gramicidin) and that the driving force for GABA was reduced in R6/2 mice, when we recorded with gramicidin. The lack of statistical significance in the driving force between WT and R6/2 when we recorded using the whole-cell configuration likely resulted from Cl^−^ dialysis between the ICS in the pipette and the cell body. Using cell-attached patch-clamp recordings, we examined the impact of depolarized *E*_GABA_ and found that GABA application increased action potential firing in symptomatic R6/2 mice, which reveals that GPe neurons have a switch in GABA polarity. In order for a switch in GABA polarity to have occurred, *E*_GABA_ must be depolarized with respect to the action potential threshold. However, we note that the depolarization of *E*_GABA_ may be exacerbated by GABA application, as it is well known that *E*_GABA_ can undergo a depolarizing shift during sustained GABA_A_R input (Kaila et al., 1993; Staley et al., 1995; Doyon et al., 2011, 2016; Raimondo et al., 2017; Pressey et al., 2023).

This discovery of excitatory GABA in GPe neurons in HD is aligned with accumulating evidence of neurophysiological alterations in GABAergic transmission and excitability in the GPe (Akopian et al., 2016; Du et al., 2016; Perez-Rosello et al., 2019; Barry et al., 2020). Notably, GPe neurons in R6/2 mice display hyperexcitability, and the blockade of GABA_A_Rs facilitates bursting activity (Akopian et al., 2016). Our finding that *E*_GABA_ is depolarized in the GPe leads us to suggest that a mechanism leading to bursting may be due to dysregulated Cl^−^ levels. Impairments in GPe neurons are well-documented in the HD, with the GPe serving as a therapeutic target. In fact, deep brain stimulation of the GPe has proven beneficial in alleviating motor and cognitive dysfunction in human HD patients (Da Cunha et al., 2015; Wojtecki et al., 2016). Given the important emerging role of GABAergic inhibition in the GPe, it will be important to consider in the future whether these aberrations in transmission and Cl^−^ regulation are specific to Type A or Type B neurons, which have distinct firing properties and outputs (Akopian et al., 2016; Hegeman et al., 2016), in order to refine further therapeutic interventions.

How the reduction of inhibition in D2 MSNs, and the emergence of excitatory GABA in the GPe, affect BG output are difficult to predict given the dynamic nature of Cl^−^. Computational modeling suggests that SNr responses to GABAergic inputs from the direct and indirect pathways are diverse, with excitatory effects attributed to Cl^−^ handling (Phillips et al., 2020). Activation of GPe neurons leads to a greater proportion of excitatory responses in downstream SNr neurons, compared with striatal neurons. GABA-mediated excitation in GPe neurons, leading to increased firing of those neurons, could exacerbate weakened inhibition through the indirect pathway and overexcitability of the thalamocortical pathway observed in HD.

### Reducing pathological levels of Cl^−^ can delay the onset of motor symptoms

In this study, we demonstrate that the use of bumetanide, a pharmacological inhibitor of NKCC1, can significantly improve motor symptoms in R6/2 until ∼12 weeks. This finding is supported by previous research employing a similar daily treatment of bumetanide in R6/2 mice (Hsu et al., 2019), which strengthens the rationale for pursuing a clinical investigation of CCCs as therapeutic targets in HD. We do note some minor differences between our study and that of Hsu et al. (2019); in their study, motor deficits appear ∼2 weeks later (∼9 weeks vs ∼7 weeks), which suggests potential variations in the progression rates among R6/2 colonies, as previously reported (Wood et al., 2008). However, our discovery that Cl^−^ regulation is impaired at an early symptomatic phase opened the possibility for CCCs to also serve as a therapeutic target in HD, as has been recently suggested (Andrews et al., 2020; Serranilla and Woodin, 2021). Based on the Cl^−^ dysregulation found specifically in D2 MSNs, we pursued a genetic approach and found that overexpressing KCC2 in this cell type alone improved motor function in HD mice. While neither therapeutic strategy restored motor symptoms completely, there are many avenues for further investigation to improve strategy and behavioral outcomes. We are currently exploring the earliest timepoint of altered Cl^−^ regulation for more precise temporal targeting and assessing the potential for recently identified small molecules KCC2 potentiators (Delpire et al., 2009; Prael et al., 2022). It is evident that Cl^−^ dysfunction and CCCs contribute to striatal synaptic dysfunction and motor impairments associated with HD and provide insight into the complex nature of synaptic mechanisms underlying the development of motor deficits in HD.
