# Supplementary figures and images for: Restoring Compromised Cl− in D2 Neurons of a Huntington’s Disease Mouse Model Rescues Motor Disability
Source: J Neurosci. 2024 Nov 5;44(50):e0215242024. doi: 10.1523/JNEUROSCI.0215-24.2024 (PMC11638812; doi:10.1523/JNEUROSCI.0215-24.2024)

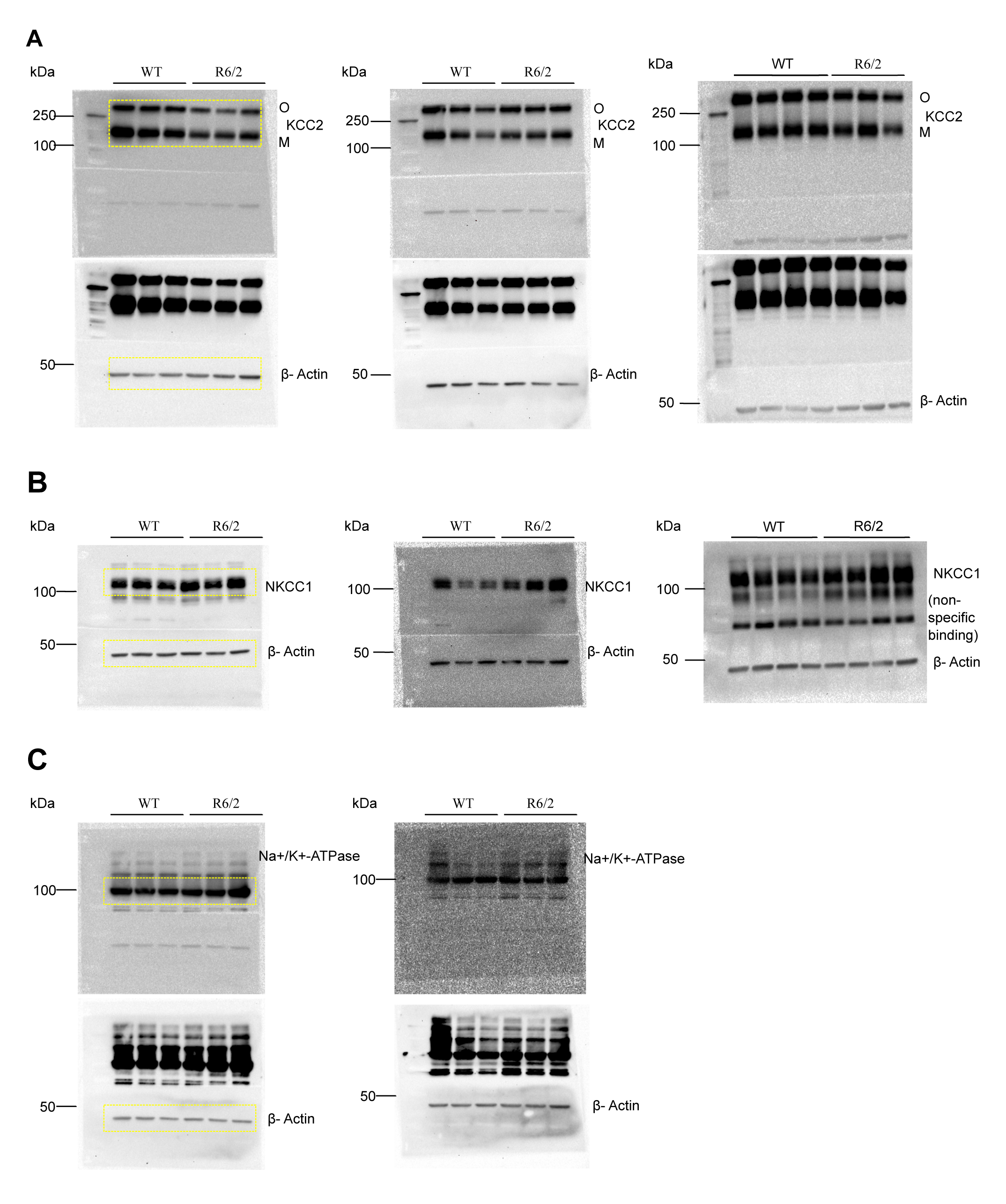

Supplement: Figure 1-1 — Full-size immunoblots related to Fig.1A-C used for quantification. O: Oligomer, M: Monomer. Yellow boxes indicate regions shown in the corresponding Figure. Download Figure 1-1, TIF file. [file jneuro-44-e0215242024-s001.tif]

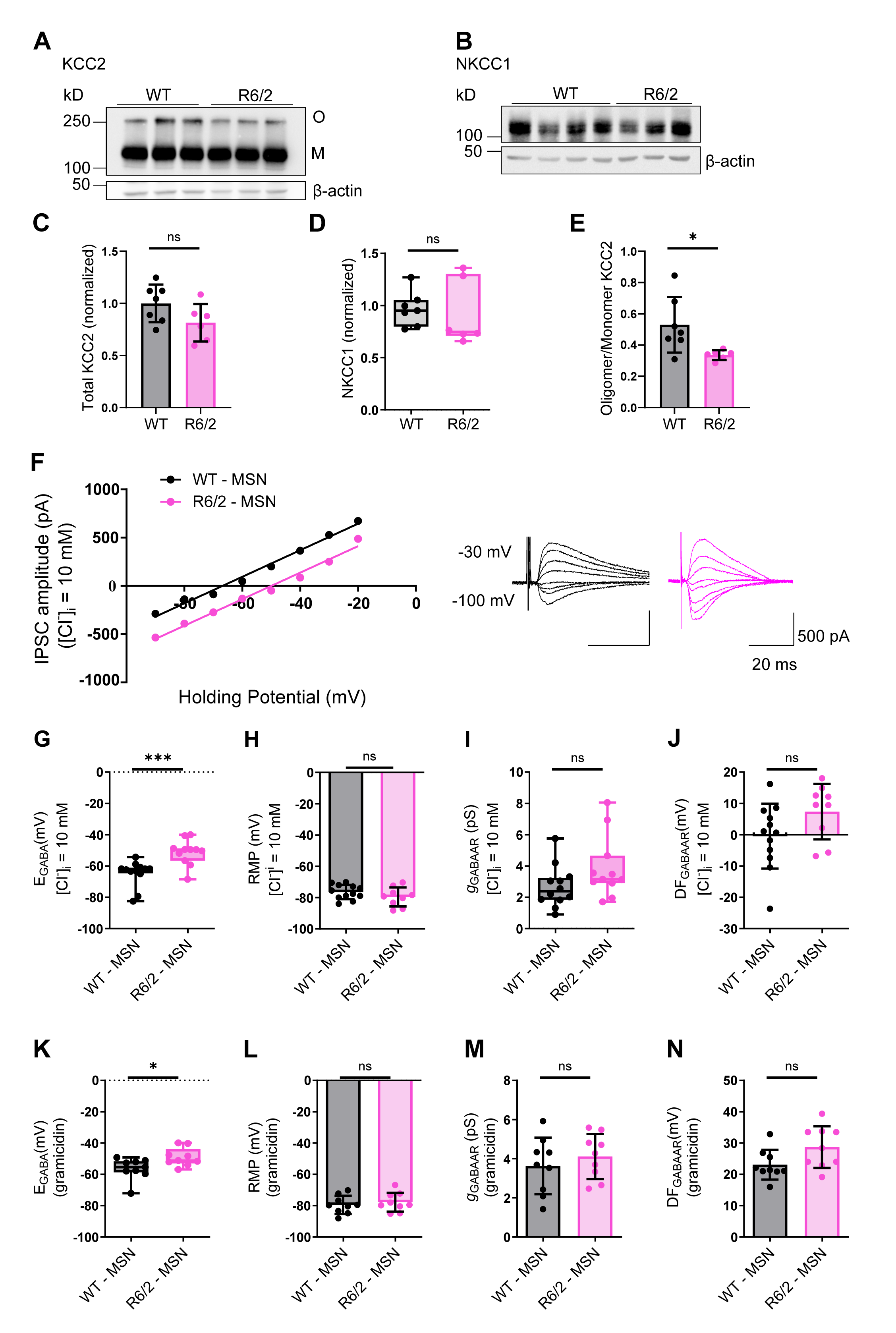

Supplement: Figure 1-2 — KCC2 expression and Cl- regulation is altered in presymptomatic R6/2. (A) Representative immunoblot image for KCC2 in protein extracts from samples of striatal lysates from presymptomatic (P35-P38) WT and R6/2. O: Oligomer, M: Monomer. (B) Representative immunoblot image for NKCC1 in protein extracts from samples of striatal lysates from presymptomatic WT and R6/2. (C) Quantification of total KCC2 (oligomer + monomer) in WT (N = 7) and R6/2 (N = 6) normalized to β-actin (P = 0.0912; Student’s unpaired t-test). Circles indicate values from individual animals. Columns represent the mean ± SD. (D) Quantification of NKCC1 in WT (N = 7) and R6/2 (N = 6) normalized to β-Actin (P = 0.3660; Mann-Whitney test). Circles indicate values from individual animals. Whiskers indicate max to min and bar indicates the median. (E) Quantification of Oligomer to Monomer ratio of KCC2 in WT (N = 7) and R6/2 (N = 6) normalized to β-Actin (P = 0.0243; Student’s unpaired t-test). Circles indicate values from individual animals. Columns represent the mean ± SD. (F) Example IV curves of representative IPSCs (right) in recorded in whole-cell patch-clamp recording configuration at different holding potentials from -100 mV to -30 mV with [Cl-]i = 10 mM from WT (black) and R6/2 (pink). (G) Summary of individual EGABA recordings with [Cl-]i = 10 mM obtained from all IV curves in WT (n = 12) and R6/2 (n = 11) (P = 0.0005; Mann-Whitney test). (H) RMP with [Cl-]i = 10 mM from WT (n = 12) and R6/2 (n = 9) (P = 0.1889; Student’s unpaired t-test). (I) Conductance through GABAAR from WT (n = 12) and R6/2 (n = 11) (P = 0.1002; Mann-Whitney test). (J) Cl- driving force through GABAAR from WT (n = 12) and R6/2 (n = 9) (P = 0.0850; Student’s unpaired t-test). (K) Similar to G but recorded in gramicidin perforated patch clamp configuration in WT (n = 9) and R6/2 (n = 9) (P = 0.0244; Mann-Whitney test). (L) Similar to H but recorded in gramicidin perforated patch clamp configuration in WT (n = 9) and R6/2 (n = [file jneuro-44-e0215242024-s002.tif]

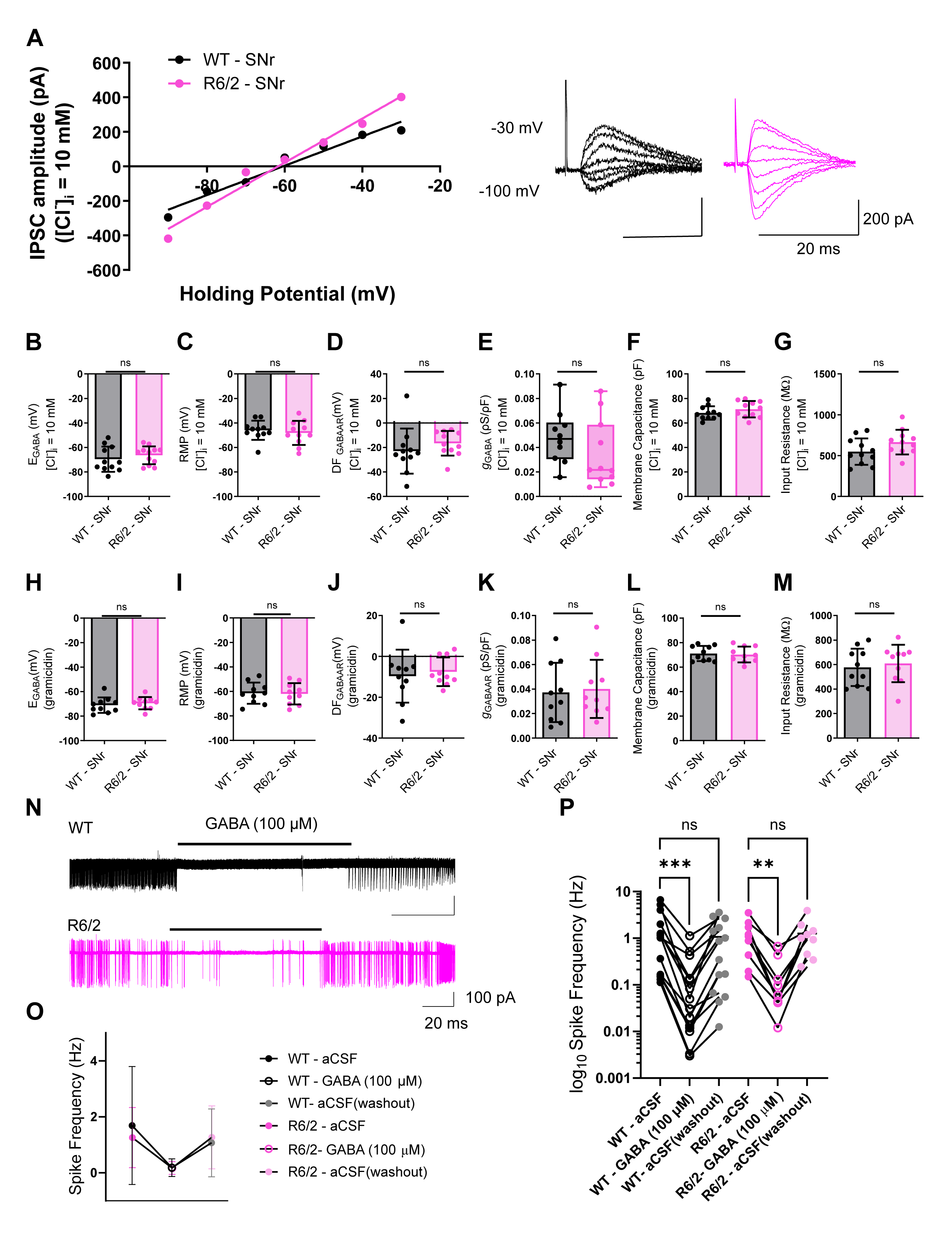

Supplement: Figure 4-3 — Cl- regulation is unaltered in the SNr of R6/2 mice. (A) Example IV curves of representative IPSCs (right) induced by electrical stimulation in the presence of glutamate blockers CNQX (10 μM) and AP-V (50 μM) at different holding potentials from -100 to -30 mV in whole-cell patch-clamp configuration with [Cl-]i = 10 mM in SNr neurons from WT (black) and R6/2 (pink). (B) Summary of individual EGABA recordings with [Cl-]i = 10 mM obtained from all IV curves in WT (n = 11) and R6/2 (n = 12) (P = 0.4015; Student’s unpaired t-test). (C) RMP with [Cl-]i = 10 mM from WT (n = 11) and R6/2 (n = 11) (P = 0.5676; Student’s unpaired t-test). (D) Cl- driving force through GABAARs with [Cl-]i = 10 mM from WT (n = 11) and R6/2 (n = 11) (P = 0.3248; Unpaired student’s t-test). (E) Conductance through GABAARs with [Cl-]i = 10 mM from WT (n = 10) and R6/2 (n = 11) (P = 0.1517; Mann-Whitney test). Whiskers indicate Min to Max and bar indicates median. (F) Membrane capacitance with [Cl-]i = 10 mM from WT (n = 11) and R6/2 (n = 11) (P = 0.2539; Student’s unpaired t-test). (G) Input resistance with [Cl-]i = 10 mM from WT (n = 11) and R6/2 (n = 11) (P = 0.0958; Student’s unpaired t-test). (H) Similar to B but recorded in gramicidin perforated patch clamp configuration from WT (n = 10) and R6/2 (n = 10) (P = 0.5374; Student’s unpaired t-test). (I) Similar to C but recorded in gramicidin perforated patch clamp configuration from WT (n = 10) and R6/2 (n = 10) (P = 0.5374; Student’s unpaired t-test). (J) Similar to D but recorded in gramicidin perforated patch clamp configuration from WT (n = 10) and R6/2 (n = 10) (P = 0.6506; Student’s unpaired t-test). (K) Similar to E but recorded in gramicidin perforated patch clamp configuration from WT (n = 10) and R6/2 (n = 10) (P = 0.7929; Student’s unpaired t-test). (L) Similar to F but recorded in gramicidin perforated patch clamp configuration from WT (n = 10) and R6/2 (n = 10) (P = 0.7666; Student’s unpaired t-test). (M) Similar to G but recorded [file jneuro-44-e0215242024-s003.tif]
